# Light‐Absorbing‐Layer‐Free Flash Lamp Annealing for High‐Throughput Fabrication of All‐Solution‐Processed Oxide Electronics

**DOI:** 10.1002/advs.77200

**Published:** 2026-08-13

**Authors:** Zetong Li, Wei He, Guangji Wang, Jingwei Zhang, Zhimin Chai, Xinchun Lu

**Affiliations:** ^1^ Beijing Key Laboratory of Atomic‐Scale Precision Manufacturing and Cross‐Scale Device Fabrication, Department of Mechanical Engineering Tsinghua University Beijing China; ^2^ State Key Laboratory of Tribology in Advanced Equipment Tsinghua University Beijing China

**Keywords:** chucks, flash lamp annealing, light‐absorbing layer, metal oxides, thin‐film transistors

## Abstract

Metal oxides exhibit high transparency across the visible spectrum, making them challenging to anneal via flash lamp annealing (FLA). Whether such materials can effectively absorb light and self‐heat remains debated. Conventional workarounds often involve light‐absorbing layers; however, these approaches introduce complexity to the fabrication process and impose limitations on device architecture. Here, we introduce a FLA strategy that enables conversion of metal oxide sol–gels into functional oxides per layer within tens of seconds on both silicon and ultra‐thin glass (UTG) substrates—without additional light‐absorbing layers—by employing a mullite chuck. Finite element analysis (FEA) results indicate that the gel‐to‐oxide transformation is initiated by direct light absorption within the metal oxides. The low thermal conductivity of mullite restricts heat dissipation, facilitating a temperature rise in the metal oxide layers with increasing pulse count and promoting complete conversion. FEA further reveals that metal oxides on UTG attain lower temperatures than those on silicon, due to greater heat dissipation into the UTG substrate. We demonstrate all‐solution‐processed thin‐film transistors and functional logic gates using FLA‐processed metal oxides. This approach shortens fabrication time by two orders of magnitude without sacrificing device performance, highlighting its potential for high‐throughput production of complex integrated circuits.

## Graphical abstract

A flash lamp annealing strategy using a mullite chuck enables rapid conversion of sol–gels to metal oxides on silicon and ultra‐thin glass without extra light‐absorbing layers. Direct light absorption initiates the transformation, and the low thermal conductivity of mullite promotes heat accumulation. All‐solution‐processed electronics fabricated in this way shorten processing time by two orders of magnitude while maintaining performance.

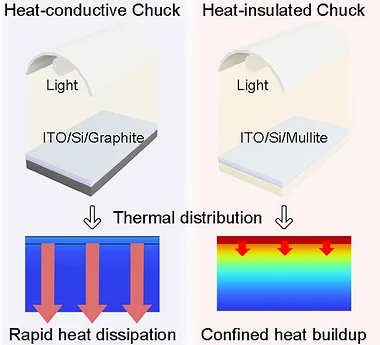

## 1 Introduction

Metal oxide‐based thin‐film transistors (MOTFTs) have garnered significant attention as enabling components for next‐generation electronic and optoelectronic devices, owing to their unique combination of high carrier mobility, excellent optical transparency, and robust environmental/thermal stability [1]. Since their integration as switching elements in active‐matrix display backplanes in 2012 [2, 3], MOTFTs have expanded into diverse cutting‐edge applications, including memory devices [4], radio‐frequency identification tags [5], logic circuits [6, 7], microprocessors [8], biosensors [9], neuromorphic computing devices [10], and beyond [11, 12]. Recently, solution‐based processes have gained widespread adoption for fabricating MOTFTs due to their inherent advantages, including compatibility with low‐cost, large‐area, and vacuum‐free processing, as well as simplicity in equipment requirements [13, 14, 15].

The solution‐based process relies on thermal annealing to trigger hydrolysis and condensation reactions, converting metal oxide sol‐gel precursors into functional oxide compounds [16, 17, 18]. However, conventional thermal annealing (CTA, Figure 1a) typically requires extended durations, often on the order of hours, to achieve complete crystallization and stoichiometric control [19], resulting in low fabrication throughput and high energy consumption. To address these limitations, alternative annealing strategies have been explored, including rapid thermal annealing (RTA) [20, 21], laser annealing (LA) [22, 23], and flash lamp annealing (FLA) [24, 25]. RTA (Figure 1b) involves rapidly heating substrates (at rates of up to 200°C s^−1^) to elevated temperatures using a high‐intensity infrared light source, maintaining them at these temperatures for short durations ranging from tens of seconds to a few minutes, and then quickly cooling them. Typically, the RTA process is conducted in controlled gas atmospheres, such as oxygen or nitrogen. While RTA reduces the total processing time to tens of minutes (including gas stabilization, heating, holding, and cooling phases), this duration remains incompatible with high‐throughput inline manufacturing. In contrast, LA employs ultrashort laser pulses (nanosecond [26, 27] to femtosecond [28, 29]) to deliver localized, high‐energy‐density heating. Nevertheless, the inherent limitation of small laser spot sizes (on the micrometer to millimeter scale [30, 31]) necessitates raster scanning for large‐area processing, thereby compromising overall fabrication efficiency. Compared to LA, FLA (Figure 1c), also termed intense pulsed light (IPL) annealing or photonic curing, offers a compelling solution by enabling large‐area (up to hundreds of millimeters) sample heating. In the FLA process, short white light pulses (microseconds to milliseconds) with a broad spectrum (200–1100 nm) and high intensity (≤100 J cm^−2^), generated by a xenon flash lamp, are delivered onto samples. These pulses are absorbed by light‐absorbing components within the samples, causing a dramatic temperature increase to several hundred degrees over a short period of time [32, 33, 34]. However, a key challenge in applying FLA to metal oxides lies in their wide bandgaps (>3 eV), which render them highly transparent across much of the visible spectrum and result in weak optical absorption. This low absorption complicates efficient annealing via the FLA process [35].

**FIGURE 1:**
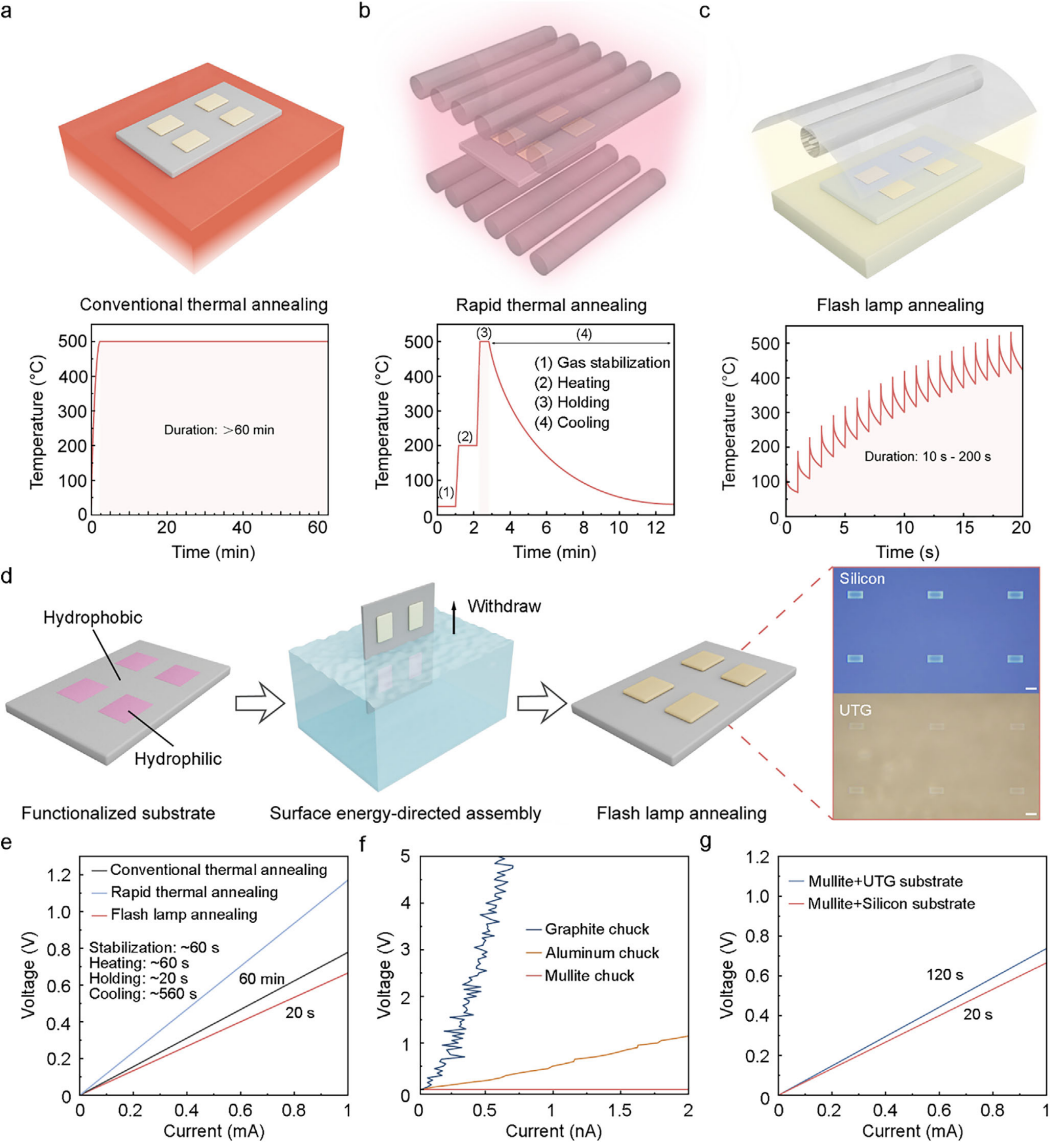
Evaluation of the feasibility of replacing conventional thermal annealing with flash lamp annealing. (a‐c) Schematic illustrations of various annealing processes along with their respective time scales. Conventional thermal annealing (a) generally necessitates several hours of processing time. Rapid thermal annealing (b) is completed within tens of minutes and involves gas stabilization, heating, holding, and cooling stages. Flash lamp annealing (c) employs intense, broad‐spectrum light pulses to deliver energy to the sample. The typical process duration ranges from tens of seconds to hundreds of seconds when multiple pulses are employed. (d) Schematic illustration of the fabrication of metal oxide patterns via a solution‐based surface energy‐directed assembly technique, followed by flash lamp annealing treatment. Included are optical images of the resultant metal oxide patterns on both silicon and ultra‐thin glass substrates. (e) Current‐voltage curves for ITO patterns subjected to conventional thermal annealing, rapid thermal annealing, and flash lamp annealing. (f) Current‐voltage curves for ITO patterns on silicon substrates annealed employing chucks with different materials. (g) Current‐voltage curves for ITO patterns on silicon and ultra‐thin glass substrates annealed with a mullite chuck.

Despite this challenge, the FLA process has been successfully used to convert sol‐gel precursors into metal oxide films in previous studies [36, 37, 38]. This conversion has often been attributed to the absorption of UV light from the broad emission spectrum of the flash lamp by the metal oxide films themselves [39, 40]. However, this interpretation remains controversial, given the relatively low proportion of UV light in the spectrum emitted by xenon flash lamps. To enhance light absorption efficiency, extrinsic light‐absorbing materials are often introduced. When sol‐gels are deposited on nontransparent substrates, such as silicon and polyimide (PI), these substrates are expected to act as light absorbers, heating the sol‐gels and facilitating their conversion into metal oxide films [41, 42, 43, 44]. Nevertheless, in other studies, additional light‑absorbing layers are still deemed necessary alongside nontransparent substrates [45]. A precise demarcation between scenarios requiring only nontransparent substrates versus those necessitating additional light‑absorbing layers remains poorly defined. In the case of transparent substrates, such as glass and polyethylene naphthalate (PEN), metal films are typically employed as additional light‑absorbing layers [46, 47, 48, 49, 50, 51]. However, these metal layers are often fabricated via vacuum‐based deposition methods, which somewhat undermines the advantage of the solution‐based fabrication of MOTFTs. Furthermore, to ensure complete chemical reactions in the dielectric and semiconducting layers, the gate electrodes must be fabricated as the bottom layer, imposing significant architectural constraints on MOTFT device design.

Here, we present a FLA process that facilitates the rapid conversion of metal oxide sol–gels into functional oxides within just tens of seconds on both silicon and ultra‐thin glass (UTG) substrates, eliminating the need for additional light‐absorbing layers. The innovation lies in leveraging an intrinsic component of the FLA system—a mullite chuck positioned beneath the substrates. Finite element analysis (FEA) simulations are conducted to elucidate how chuck material selection governs the gel‐to‐oxide conversion process across different substrates. Employing a solution‐based surface energy‐directed assembly (SEDA) technique, we fabricate a diverse array of metal oxide patterns, including indium tin oxide (ITO), aluminum oxide (Al_2_O_3_), and indium oxide (In_2_O_3_). These patterns are then subjected to FLA treatment, and their electrical properties are systematically assessed. Moreover, we successfully realize all‐solution‐processed MOTFTs and functional logic gates, highlighting the immense potential of this approach for the high‐rate fabrication of complex integrated circuits.

## 2 Results and Discussion

### 2.1 FLA of Metal Oxide Patterns

To assess the viability of substituting CTA with the FLA process, metal oxide patterns are fabricated using the SEDA technique [52, 53, 54], followed by FLA treatment, as illustrated in Figure 1d. Optical images in Figure 1d present the resultant metal oxide patterns on both silicon and UTG substrates. In the SEDA process, functionalized substrates with patterned hydrophilic regions are withdrawn from metal oxide precursor solutions. As this occurs, the solutions are selectively drawn onto the high‐surface‐energy hydrophilic patterned regions, while the low‐surface‐energy hydrophobic non‐patterned regions remain devoid of coating. Upon solvent evaporation, metal oxide wet gel patterns are formed, replicating the geometry of the hydrophilic patterns. Subsequently, the wet gel patterns undergo annealing via the FLA process, which promotes hydrolysis and condensation reactions, transforming the gels into solid metal oxide patterns. During FLA, the samples are exposed to pulsed light generated at 1100 V, with a pulse width of 10 ms, a pulse interval of 1 s, and varying pulse counts. The resistivity of FLA‐processed ITO patterns subjected to 20 pulses (approximately 20 s) is characterized using the four‐point probe method (see Note S1). A representative current−voltage (*I*−*V*) curve is shown in Figure 1e, from which a resistivity of 4.3 × 10^−3^ Ω cm is extracted. This value is either comparable to or even lower than those reported for ITO films fabricated and annealed by other methods (see Note S2). The combination of a short annealing time of approximately 20 s and low resistivity underscores the efficiency and reliability of the FLA process. For comparison, the electrical properties of ITO patterns annealed via CTA and RTA are also evaluated (Figure 1e). These processes necessitate annealing times on the order of several hours and tens of minutes, respectively. Notably, the resistivity of ITO patterns processed by FLA for only 20 s is slightly lower than that of those treated with CTA at 500°C for 60 min (6.3 × 10^−3^ Ω cm) and RTA for ∼12 min (8.7 × 10^−3^ Ω cm). When the annealing time is fixed at 20 s, the resistivity of CTA‐treated samples is approximately 6 times higher than that of the FLA‐treated sample (see Note S3). These findings further substantiate the feasibility of replacing conventional thermal annealing with the FLA process.

It is crucial to emphasize that the success of the FLA process strongly depends on the choice of the chuck material. Under an identical pulse number of 20, the resistivities of annealed ITO patterns on silicon substrates can exhibit variations spanning several orders of magnitude, depending on the type of chuck employed (Figure 1f). Specifically, when mullite, graphite, and aluminum chucks are employed, the resulting resistivities are 4.3 × 10^−3^ Ω cm, 5.9 × 10^4^ Ω cm, and 2.8 × 10^3^ Ω cm, respectively. Moreover, the substrate type also influences the FLA outcome: a longer processing time is required for ITO films on UTG substrates compared to those on silicon substrates to achieve comparable resistivity levels (Figure 1g). These findings highlight the need for further exploration of the fundamental mechanisms governing FLA.

### 2.2 Role of Chucks and Substrates in the FLA Process

In contrast to previous reports, this work demonstrates that ITO patterns on both silicon and UTG substrates can be successfully annealed via FLA without the need for additional light‐absorbing layers, provided a mullite chuck is used. To elucidate the role of the chuck in the gel‐to‐oxide conversion process, we simulate the temperature distribution across the ITO/substrate/chuck stack (Figure 2a) during FLA using FEA. Simulation details are provided in the Methods Section and Notes S4 and S5. Figures 2b and c show the simulated temperature evolution profiles of the top surface of the ITO film and the bottom of the chuck, respectively, during 20 FLA light pulses with silicon substrates placed on different chucks. The temperature rises rapidly during each pulse and then drops quickly before the next pulse. Peak‐temperature colormaps of the stack structures after the 20^th^ pulse are shown in Figure 2d. When a mullite chuck is used, the ITO film surface temperature exceeds 530°C, whereas with aluminum or graphite chucks, it remains below 200°C. This significant temperature difference is attributed to the distinct thermal conductivities of the chuck materials: 0.4 W m^−1^ K^−1^ for mullite, compared to 234 W m^−1^ K^−1^ for aluminum and 140 W m^−1^ K^−1^ for graphite. Under irradiation, both the ITO film and silicon substrate absorb incident light and heat up. The low thermal conductivity of mullite restricts heat dissipation, as evidenced by the nearly constant temperature of 25°C at the chuck bottom. This enables heat to accumulate within the ITO/silicon stack with an increasing number of pulses, elevating the temperatures of the ITO film and facilitating a more complete gel‐to‐oxide conversion, which accounts for the low resistivity of the resulting ITO patterns. In contrast, aluminum and graphite chucks function as efficient heat sinks due to their high thermal conductivity. Heat is rapidly drawn away from the ITO film and substrate, leading to a noticeable temperature increase at the chuck bottom as the number of pulses rises. Such heat dissipation lowers the peak temperature in the ITO film, resulting in an incomplete gel‐to‐oxide conversion and thus higher resistivity in the ITO patterns. This phenomenon of suppressed peak temperature caused by rapid heat dissipation has been previously observed in the FLA processing of copper nanoparticle pastes printed on high‐thermal‐conductivity silicon substrates, where the peak temperature of the copper layer was significantly limited [55].

**FIGURE 2:**
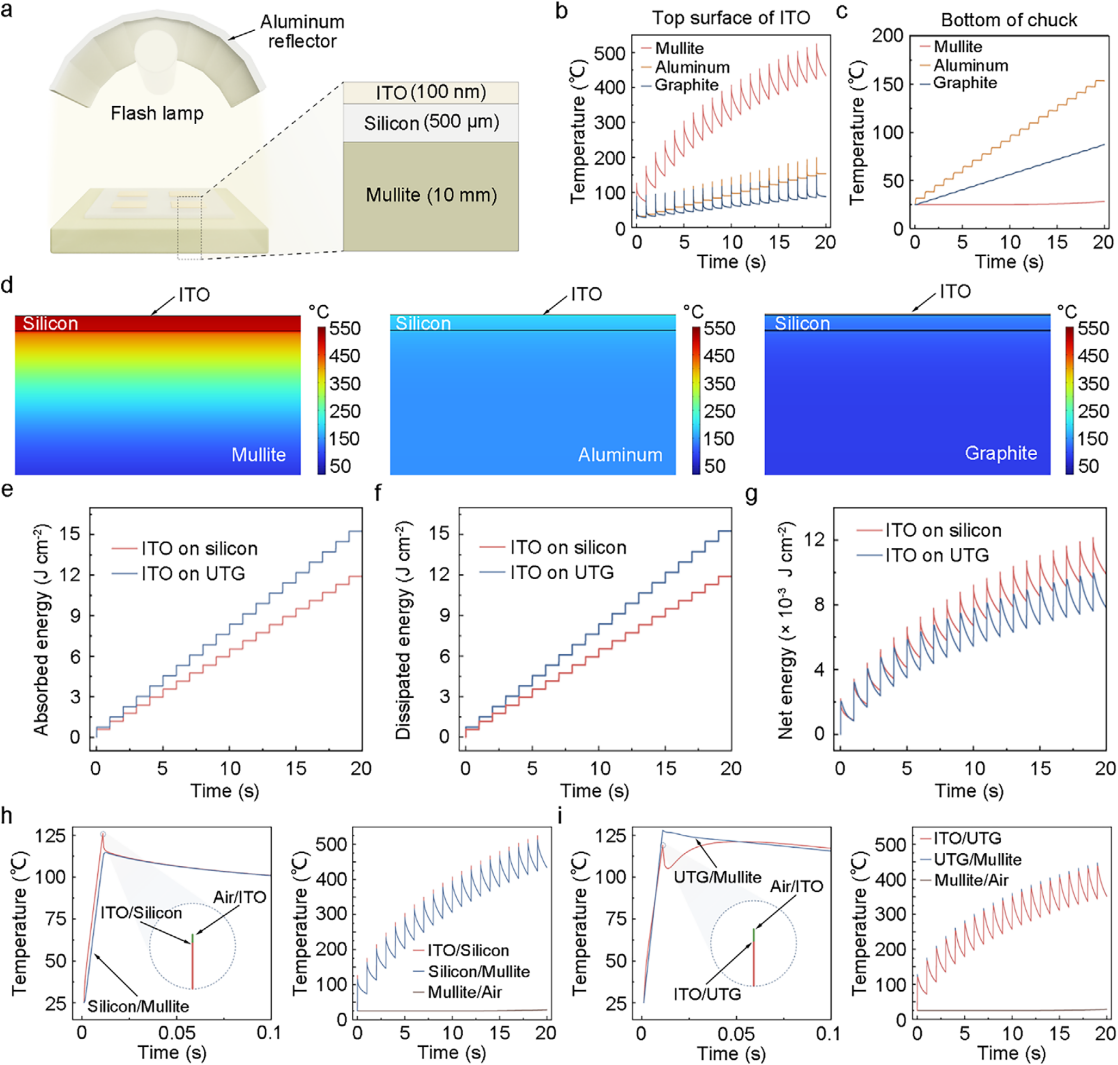
Role of chucks and substrates in the FLA process. (a) Schematic diagram of the FLA setup with an ITO/UTG/mullite stack structure. (b, c) Simulated temperature evolution of the top surface of the ITO film (b) and the bottom of the chuck (c) during 20 light pulses with silicon substrates placed on different chucks (mullite, aluminum, and graphite). (d) Peak‐temperature colormaps of different stack structures after the 20^th^ pulse. (e) Light energy absorbed by the ITO films on silicon and UTG substrates over 20 pulses. (f) Heat dissipation from the ITO films to the underlying substrates and to the ambient air over 20 pulses. (g) Net energy retained in the ITO films on silicon and UTG substrates over 20 pulses. (h) Temperature evolution at the air/ITO, ITO/silicon, silicon/mullite, and mullite/air interfaces during a single pulse (left) and 20 pulses (right). The inset in the left panel shows that the temperature at the air/ITO interface is slightly higher than that at the ITO/silicon interface. (i) Temperature evolution at the air/ITO, ITO/UTG, UTG/mullite, and mullite/air interfaces during a single pulse (left) and 20 pulses (right).

This work further reveals that ITO patterns on the silicon substrate undergo annealing more efficiently than those on the UTG substrate, as evidenced by the shorter annealing time required to achieve comparable resistivity. Finite element simulations indicate that the ITO film in the ITO/silicon/mullite stack absorbs 5.3% of the incident light energy (11.2 J cm^−2^) per pulse (Figure 2e). Despite its limited light absorption, the ITO film, with a thin thickness of only 100 nm, reaches a surface temperature of approximately 125°C due to its low thermal mass (Figure 2h). The temperature at the top surface of the ITO film is slightly higher than that at the bottom, indicating that heat conduction occurs from the ITO film into the substrate. This result also implies that the thermal energy in the ITO film originates primarily from direct light absorption rather than from heat transfer from the substrate. The robustness of this conclusion has been verified by considering uncertainties in the input parameters as well as their temperature dependence (see Notes S6 and S7). By subtracting the energy dissipated to the substrate (Figure 2f) from the absorbed energy, the net energy retained in the ITO film can be determined (Figure 2g). This net energy accumulates with increasing pulse number, leading to a high surface temperature of approximately 530°C after 20 pulses. An auxiliary thermal evaluation using temperature‐indicating strips confirms that the temperature increases monotonically with the pulse number (Figure S9), providing direct evidence for the heat accumulation effect. In contrast, the ITO film in the UTG‐based stack absorbs 6.8% of the incident energy per pulse (Figure 2e)—a higher absorption than in the silicon‐based stack. However, because the UTG substrate is highly transparent in the visible light regime, it absorbs much less incident light than the silicon substrate. Consequently, more heat is dissipated from the ITO film into the UTG substrate, resulting in a lower net energy retained in the ITO film (Figure 2g). Under the same 20‐pulse treatment, the ITO film on UTG reaches a peak temperature of only about 430°C (Figure 2i), which is lower than that on silicon. Notably, the function of the mullite chuck differs between the two stack configurations. In the ITO/silicon/mullite stack, where the ITO film and silicon substrate absorb most of the energy, the mullite chuck acts primarily as a thermal insulator, minimizing heat dissipation. In the ITO/UTG/mullite stack, however, the mullite chuck absorbs the majority of the incident energy and serves as a hot plate, supplying energy to the UTG substrate and maintaining the high temperature of the ITO film (Figure 2i).

### 2.3 Electrical Properties of Various Metal Oxides Processed Using FLA

The electrical properties of various metal oxides processed via FLA are systematically evaluated. Figure 3a presents the resistivity of ITO patterns fabricated on both silicon and UTG substrates after annealing on different chucks under varying numbers of light pulses. For each annealing condition, resistivity values are measured for at least 10 individual patterns. As anticipated, ITO patterns annealed using the aluminum chuck exhibit resistivities up to six orders of magnitude higher than those treated with the mullite chuck. This outcome can be attributed to the enhanced heat dissipation caused by the aluminum chuck, which limits the achievable temperature within the ITO patterns and consequently leads to incomplete gel‐to‐oxide conversion, as discussed earlier. The effect is even more pronounced with a graphite chuck, where significant heat loss results in resistivities on the order of thousands of Ω cm even after 200 pulses. In contrast, when the mullite chuck is employed, the resistivity of the ITO patterns on the silicon substrate can decrease to as low as 1.0 × 10^−2^ Ω cm after only 10 light pulses, owing to reduced heat dissipation. The resultant heat accumulation raises the temperature of the ITO patterns to approximately 390°C, which is sufficient to drive the gel‐to‐oxide conversion. This conversion is evidenced by a pronounced thickness reduction from about 142.4 nm for the as‐prepared wet gel patterns to 32.7 nm for the metal oxide patterns after 20 pulses (Figure S10), as well as a decrease in surface roughness from 1.12 nm to 0.30 nm over the same treatment (Figure S11). As the number of pulses increases, the temperature within the ITO patterns continues to rise, enhancing crystallization and further reducing the resistivity, thickness, and surface roughness. After 200 pulses, the resistivity and thickness reach 2.2 × 10^−3^ Ω cm and 29.5 nm, respectively, while the surface roughness decreases to 0.17 nm after 100 pulses. This progressive crystallization is supported by X‐ray photoelectron spectroscopy (XPS) analysis (Figure 3b; Figure S12), which shows an increasing proportion of metal‐oxygen‐metal (M‐O‐M) bonding state alongside a decrease in metal hydroxide (M‐OH) content and oxygen vacancies. Further evidence of improved crystallinity comes from X‐ray diffraction (XRD) data (Figure 3c), where the intensity of the characteristic ITO peak at 2*θ* = 30.5° increases with the number of pulses. Additionally, two‐dimensional grazing‐incidence wide‐angle X‐ray scattering (2D GIWAXS) measurements (Figure 3d) reveal a transition from a diffuse halo at 10 pulses—indicating low crystallinity—to ring‐like scattering features after 40 and 100 pulses, signifying the development of a polycrystalline structure in the ITO films [56, 57].

**FIGURE 3:**
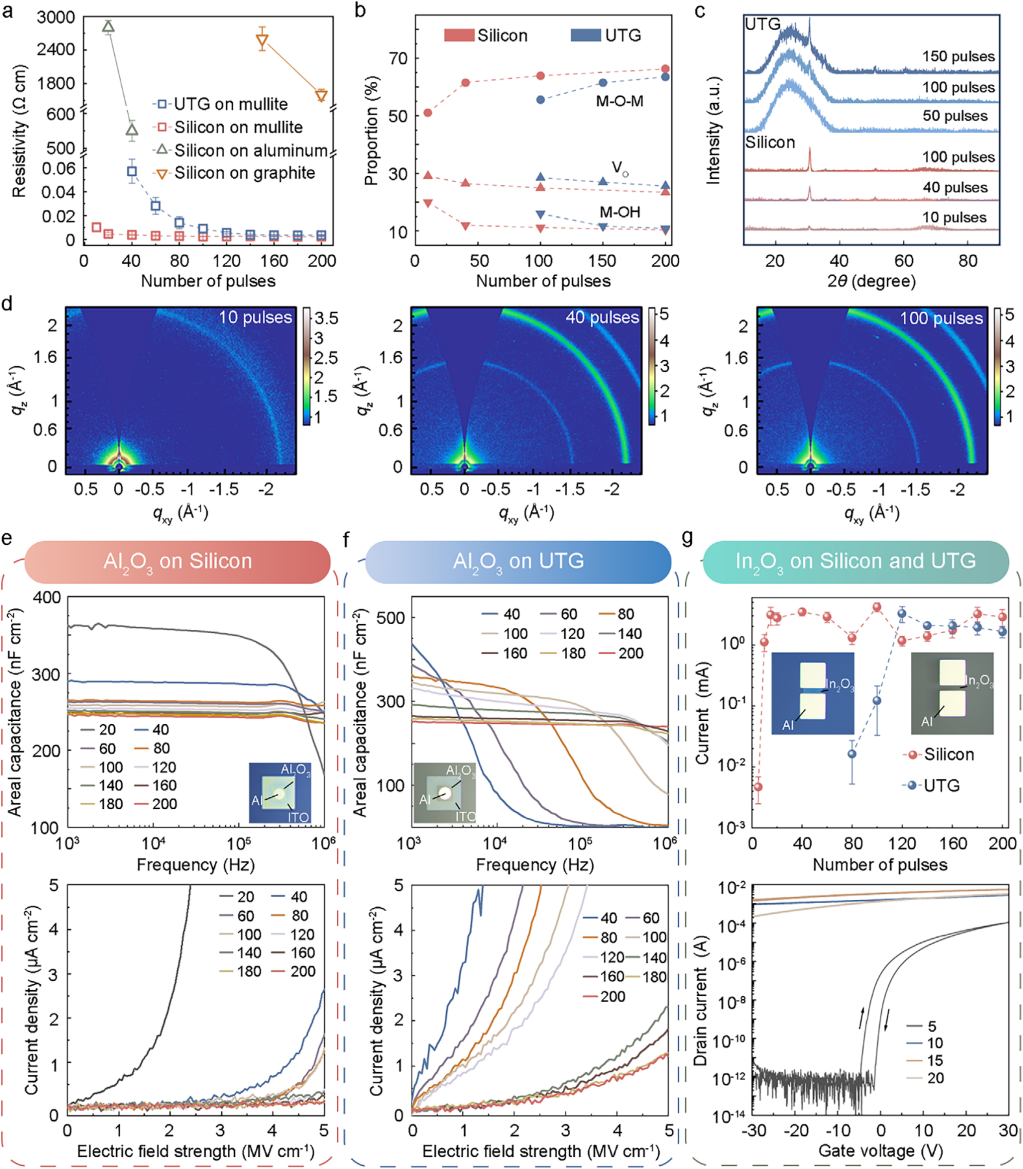
Electrical properties of various metal oxides processed using FLA. (a) Resistivity of ITO patterns fabricated on silicon and UTG substrates as a function of pulse number, using different chuck materials. (b) Evolution of M─O─M, M─OH, and oxygen vacancy (V_O_) proportions derived from XPS analysis with increasing pulse number. (c) XRD patterns of ITO films on silicon and UTG substrates annealed with different numbers of pulses. (d) 2D GIWAXS patterns of ITO films on silicon substrates annealed with varying numbers of pulses. (e, f) Areal capacitance versus frequency (top) and leakage current density versus electric field strength (bottom) for Al_2_O_3_‐based capacitors fabricated on silicon (e) and UTG (f) substrates. Insets illustrate the MIM capacitor structure with ITO bottom electrodes and aluminum top electrodes. (g) Top: current through In_2_O_3_ patterns on silicon and UTG substrates under 10 V bias after annealing at different pulse numbers. Insets illustrate aluminum electrodes fabricated atop In_2_O_3_ patterns. Bottom: transfer characteristics of In_2_O_3_‐based TFTs on silicon substrates under varying pulse numbers.

As compared to those on silicon substrates, ITO patterns fabricated on UTG substrates require more light pulses to achieve comparable resistivity. Specifically, 20 and 120 pulses are needed for ITO patterns on silicon and UTG substrates, respectively, to reach resistivities around 4 × 10^−3^ Ω cm. According to simulation results, the inferior conductivity of ITO patterns on UTG substrates stems from their lower processing temperatures under the same number of pulses relative to those on silicon. Due to these lower temperatures, ITO patterns on UTG undergo less complete crystallization, as reflected in a lower proportion of M‐O‐M bonding states observed in XPS analysis (Figure 3b; Figure S12). Furthermore, distinct XRD diffraction peaks only emerge after more than 100 pulses for ITO films on UTG (Figure 3c), whereas those on silicon substrates exhibit such features after just 40 pulses.

The dielectric and insulating properties of FLA‐processed Al_2_O_3_ patterns on silicon and UTG substrates are also characterized. For this purpose, metal‐insulator‐metal (MIM) capacitors are constructed, with ITO patterns (150 µm × 150 µm) serving as the bottom electrodes, Al_2_O_3_ patterns (70 µm × 70 µm) as the insulators, and aluminum pads (50 µm diameter) as the top electrodes. The thickness of the Al_2_O_3_ patterns decreases with increasing pulse number (Figure S13), stabilizing at approximately 20.2 nm on silicon substrates and 26.5 nm on UTG substrates. The surface roughness also declines with more pulses (Figure S14). Figure 3e presents the areal capacitance versus frequency and the leakage current density versus applied electric field strength for capacitors fabricated on silicon substrates. Those with Al_2_O_3_ annealed under 20 pulses show the highest areal capacitance at 1 kHz. The corresponding dielectric constants, derived from the capacitance values, are approximately 12.2 (Figure S15), surpassing the typical value of 9 for Al_2_O_3_. This elevated capacitance and dielectric constant at low pulse numbers arise from the incomplete conversion of M─OH to M─O─M bonds due to the low processing temperature (Figure S16). Residual hydroxyl groups promote the absorption of water molecules, which possess a high dielectric constant of 80, thereby increasing the overall dielectric constant and capacitance [58, 59]. Furthermore, the slow polarization response of water molecules at high frequencies causes the capacitance to decline with increasing frequency in samples subjected to 20 and 40 pulses [58]. As the pulse number increases, more complete M─O─M bonding reduces water absorption, leading to lower areal capacitance and weaker frequency dependence. The improved bond formation also accounts for the reduction in leakage current density from 2.67 µA cm^−2^ at 5 MV cm^−1^ after 40 pulses to 0.10 µA cm^−2^ under the same field strength after 200 pulses. Compared to Al_2_O_3_ on silicon, patterns on UTG substrates exhibit higher areal capacitance and greater leakage current under the same pulse counts (Figure 3f; Figure S17). This can be attributed to less complete conversion of M─OH to M─O─M bonds (Figure S16), resulting from the lower temperature achievable in the Al_2_O_3_ patterns on UTG substrates.

The electrical properties of FLA‐processed In_2_O_3_ patterns on silicon and UTG substrates are characterized as well. For each annealing condition, at least 10 individual In_2_O_3_ patterns are measured. Aluminum pads (100 µm × 100 µm) spaced 20 µm apart are deposited on the annealed In_2_O_3_ patterns (80 µm × 40 µm). Figure 3g shows the current flowing through the In_2_O_3_ patterns under an applied voltage of 10 V. The current increases with the number of pulses, which is attributed to the enhanced crystallization of In_2_O_3_ (Figures S18–S21). Similar to the trends observed for ITO and Al_2_O_3_, the In_2_O_3_ patterns on silicon substrates exhibit higher current levels than those on UTG, consistent with their higher degree of crystallinity. Bottom‐gate top‐contact TFTs are fabricated using silicon substrates as the gate electrodes, silicon dioxide films as the gate dielectric layers, In_2_O_3_ patterns as the semiconducting channels, and aluminum pads as the source/drain electrodes. The transfer characteristics of these TFTs are shown in Figure 3g. TFTs annealed with 5 pulses exhibit switching behavior with a high on/off ratio exceeding 10^8^. However, as the pulse number increases to 10 or more, the TFTs lose their switching capability. This transition is attributed to the increased proportion of M─O─M bonding, which promotes the formation of conductive pathways and renders the In_2_O_3_ layer more conductor‐like.

### 2.4 Fabrication and Characterization of FLA‐Processed MOTFTs

MOTFTs with a top‐gate top‐contact configuration are fabricated, consisting of sequential In_2_O_3_/ITO/Al_2_O_3_/ITO layers, with a channel length and width of 20 µm and 80 µm, respectively (Figure 4a–c). The dimensions and relative positions of each layer, along with the fabrication procedure, are detailed in Note S13. All layers are annealed via the FLA process using the same number of pulses for each. Cross‐sectional transmission electron microscope (TEM) images of MOTFTs processed with 60 pulses on both silicon and UTG substrates are shown in Figure 4d,e, in which each functional layer is clearly distinguishable. The layers fabricated on UTG substrates exhibit noticeably greater thickness than those on silicon, consistent with AFM‐based thickness measurements. A cumulative effect of the FLA process is also observed. Although each layer receives the same number of pulses, the microstructure of the ITO in the fourth layer (gate electrode) differs significantly from that in the second layer (source/drain electrodes). The second‐layer ITO is subjected to three intermittent 60‐pulse treatments, whereas the fourth‐layer ITO undergoes only one 60‐pulse treatment. As a result, the former shows substantially higher crystallinity and larger grain size, while the latter contains a partial amorphous phase. It should be noted that a single 60‐pulse treatment is insufficient to fully crystallize ITO on UTG substrates. Crystallization remains in the nucleation and slow‐growth stage, leading to only minor microstructural differences between the second‐ and fourth‐layer ITO patterns on UTG.

**FIGURE 4:**
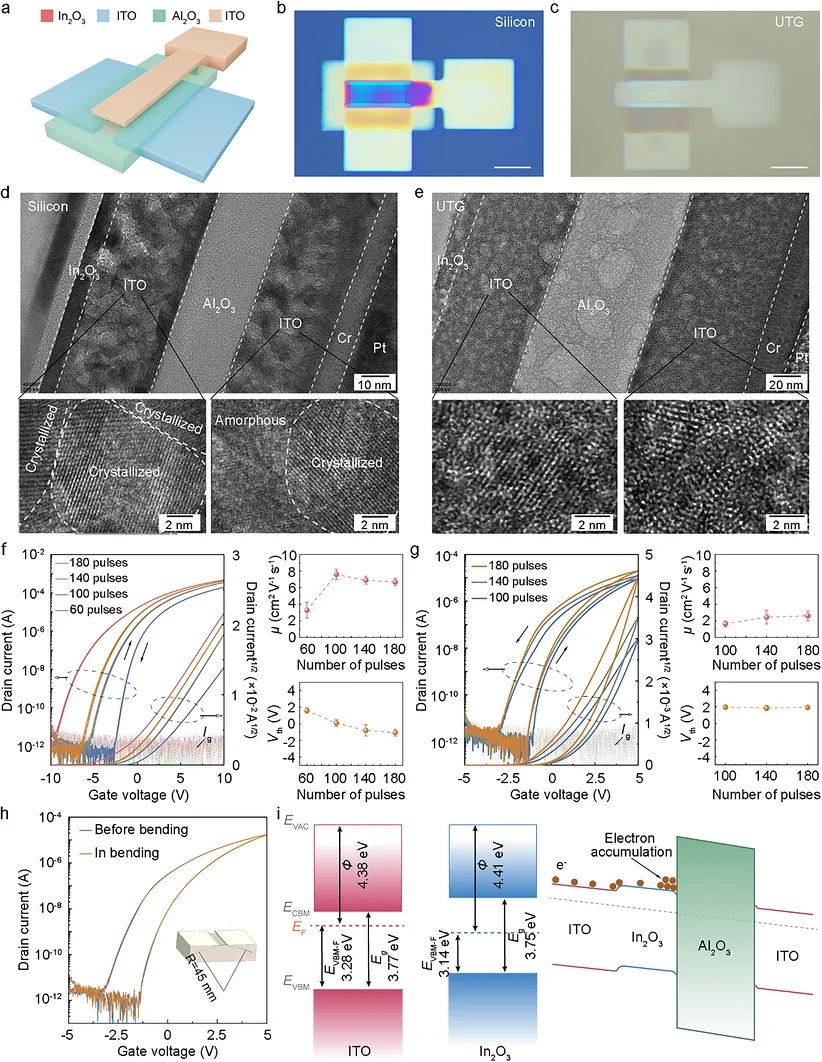
Fabrication and electrical characterization of FLA‐processed MOTFTs. (a) Schematic illustration of MOTFTs with a top‐gate top‐contact configuration, consisting of sequential In_2_O_3_/ITO/Al_2_O_3_/ITO layers. (b, c) Optical images of MOTFTs fabricated on silicon (b) and UTG (c) substrates. Scale bar: 50 µm. (d, e) Cross‐sectional TEM images of MOTFTs processed with 60 pulses on both silicon (d) and UTG (e) substrates. (f, g) Transfer characteristics, mobilities, and threshold voltages of MOTFTs fabricated on silicon (f) and UTG (g) substrates processed with different numbers of pulses. (h) Transfer characteristics of a representative UTG‐based MOTFT before and during bending. (i) Measured values of *Φ*, *E*_VBM‐F,_ and *E*_g_ for In_2_O_3_ and ITO films processed with 100 pulses on UTG substrates, along with the energy band diagram of the ITO/In_2_O_3_/Al_2_O_3_ heterojunction.

The transfer characteristics of MOTFTs processed with 20, 60, 100, 140, and 180 pulses are shown in Figure 4f,g. Devices fabricated on silicon substrates and treated with 20 pulses exhibit severe leakage current (Figure S23), attributed to the incomplete conversion of M─OH to M─O─M bonds in the Al_2_O_3_ dielectric layers. As the pulse number increases, more complete bond conversion occurs, reducing the leakage current to below 20 picoamps. The saturation mobilities (*μ*) for MOTFTs treated with 60, 100, 140, and 180 pulses are 3.28 ± 0.95, 7.59 ± 0.63, 6.90 ± 0.47, and 6.67 ± 0.44 cm^2^ V^−1^ s^−1^, respectively, with corresponding threshold voltages (*V*_th_) of 1.61 ± 0.32, 0.10 ± 0.41, −0.79 ± 0.63, and −1.06 ± 0.42 V. Each data set was obtained from 100 devices per condition. The initial increase in *μ* from 60 to 100 pulses is primarily attributed to the enhanced crystallization of the In_2_O_3_ channel, which improves electron percolation pathways and thereby facilitates efficient electron transport. As the pulse number further increases, *μ* exhibits a slight downward shift. This minor performance degradation can be plausibly attributed to intensified electron scattering during fast electron transportation. The improved electron transport requires a more negative gate voltage to deplete the channel and turn off the device, contributing to the negative shift in threshold voltage. The decrease in the dielectric constant of Al_2_O_3_ with increasing pulse number also promotes this negative shift. Similarly, MOTFTs on UTG substrates show a rise in saturation mobility with pulse number, reaching 1.64 ± 0.35, 2.45 ± 0.72, and 2.61 ± 0.45 cm^2^ V^−1^ s^−1^ at 100, 140, and 180 pulses, respectively. This increase in mobility can also be attributed to the enhanced crystallization of the In_2_O_3_ patterns. However, in contrast to MOTFTs fabricated on silicon substrates, those on UTG exhibit minimal threshold voltage shift, with values of 1.97 ± 0.12, 1.85 ± 0.29, and 1.93 ± 0.12 V under 100, 140, and 180 pulses, respectively. UTG‐based MOTFTs also display more positive threshold voltages than their silicon counterparts. The minimal threshold voltage shift and more positive threshold voltage are likely due to the higher dielectric constant of Al_2_O_3_ on UTG substrates, which strengthens gate control and facilitates more complete channel depletion. As noted earlier, the higher dielectric constant of Al_2_O_3_ on UTG originates from its greater hydroxyl group content, which promotes water adsorption. During a forward voltage sweep, water molecule polarization induces positive charge accumulation near the In_2_O_3_/Al_2_O_3_ interface. Upon reverse sweeping, slow depolarization mimics an additional positive gate bias, resulting in counterclockwise hysteresis. As summarized in Table S7, the TFTs in this work exhibit electrical characteristics comparable or superior to those fabricated using CTA, while reducing processing time by up to two orders of magnitude.

The electrical stability of the MOTFTs is investigated using the positive bias stress (PBS) test (Figure S25). The MOTFT fabricated on the silicon substrate demonstrates excellent PBS stability, with a threshold voltage shift of only 0.08 V after 3600 s of stress. In contrast, the MOTFT fabricated on the UTG substrate exhibits inferior PBS stability, with a larger threshold voltage shift of 0.34 V. The positive threshold voltage shift observed for both MOTFTs is attributed to electron trapping at the In_2_O_3_/Al_2_O_3_ interface. The more pronounced positive shift for the MOTFT on UTG can be ascribed to the higher hydroxyl group content in the Al_2_O_3_ gate dielectric on UTG. These hydroxyl groups act as electron traps, capturing electrons within the In_2_O_3_ channel and thereby requiring a larger gate voltage to induce sufficient channel carriers. This results in an enhanced positive shift of the threshold voltage. Furthermore, both MOTFTs demonstrate excellent environmental stability after six months of storage in ambient air, confirming their robustness against long‐term atmospheric exposure (Figure S26). Mechanical stability is evaluated through flexible bending tests on UTG‐based MOTFTs using a custom fixture (Figure 4h). The transfer characteristics of a representative MOTFT measured before and during bending at a radius of 45 mm (corresponding to a strain of 0.2%) show negligible deviation, confirming high mechanical stability. Moreover, the MOTFT retains its electrical performance after 1000 repeated bending cycles at the same radius (Figure S27), further demonstrating its high mechanical endurance.

To assess the suitability of ITO as source/drain and gate electrodes, the energy band structures of In_2_O_3_ and ITO are analyzed. Ultraviolet photoelectron spectroscopy (UPS) is used to determine the work function (*Φ*) and the energy difference between the valence band maximum and the Fermi level (*E*_VBM‐F_) for both In_2_O_3_ and ITO. Meanwhile, a UV–vis spectrophotometer is employed to measure the optical bandgap (*E*_g_). Detailed characterization methods are provided in Note S17. Figure 4i and Table S8 summarize the measured values of *Φ*, *E*_VBM‐F_, and *E*_g_ for films processed with 100 pulses on UTG substrates, along with the corresponding energy band diagram of the ITO/In_2_O_3_/Al_2_O_3_ heterojunction. Given that the work function of ITO is lower than that of In_2_O_3_, the alignment of Fermi levels at the ITO/In_2_O_3_ interface induces electron transfer from ITO to In_2_O_3_. This electron transfer establishes an Ohmic contact at the ITO/In_2_O_3_ interface, thereby facilitating efficient electron injection from the ITO electrode into the In_2_O_3_ channel. Additionally, downward band bending at the In_2_O_3_/Al_2_O_3_ interface forms a two‐dimensional (2D) potential well that effectively confines electrons and enhances the conductivity of the channel. The simultaneous formation of an Ohmic contact and a 2D potential well provides strong evidence supporting the suitability of ITO for use as source/drain and gate electrodes in these devices.

### 2.5 Logic Gate Circuits Integrated With FLA‐Processed MOTFTs

To demonstrate the applicability of the FLA process in fabricating functional circuits, we fabricate various logic gates consisting of FLA‐processed MOTFTs, including NOT, NOR, NAND, and AND gates, as shown in Figure 5a. The integrated MOTFTs feature a channel length of 5 µm and a channel width of 40 µm. The NOT gate comprises two MOTFTs, the NOR and NAND gates each contain three, and the AND gate incorporates five MOTFTs. The dynamic response of each circuit is measured using square‐wave input signals (*V*_IN_) with an amplitude of 2 V and a supply voltage (*V*_DD_) of 2 V. The output logic state is defined as “1” when the output voltage (*V*_out_) equals *V*_DD_, and “0” when *V*_out_ is 0 V. The circuit diagrams and corresponding input‐output responses are presented in Figure 5b,c. In the NOT gate, a logic “1” is output at an input voltage of −2 V, and a logic “0” at 2 V. In the NOR gate, a logic “1” is generated only when both inputs *V*_IN1_ and *V*_IN2_ are −2 V, while a logic “0” is output when either *V*_IN1_ or *V*_IN2_ reaches 2 V. For the NAND gate, a logic “1” results when either *V*_IN1_ or *V*_IN2_ is −2 V, and a logic “0” when both inputs are 2 V. In the AND gate, a logic “1” is output only when both *V*_IN1_ and *V*_IN2_ are 2 V, whereas a logic “0” is produced when either input is −2 V. These input‐output relationships agree well with the expected logical functions, confirming the successful implementation of the fabricated logic circuits. After six months of ambient storage, all logic gates retain their correct logic functionality with negligible degradation in output characteristics, further highlighting the exceptional long‑term reliability of the FLA‑processed devices (Figure S30). Furthermore, the NOT gate exhibits nearly delay‐free inversion at 1 kHz and retains clear signal inversion up to 4 kHz (Note S19), underscoring the high reliability of the FLA‐processed devices.

**FIGURE 5:**
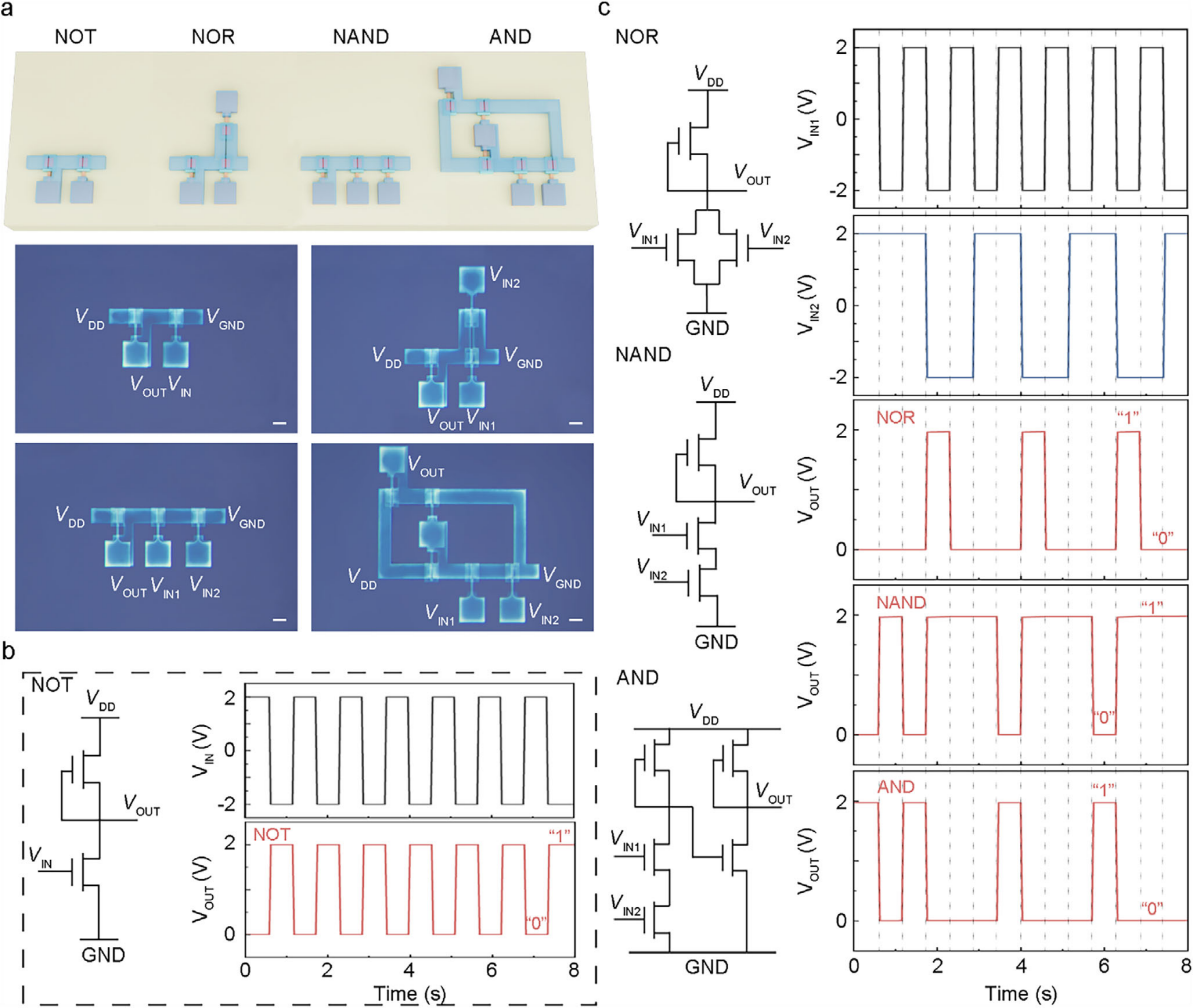
Implementation of logic gates based on FLA‐processed MOTFTs. (a) Schematic illustrations and optical micrographs of NOT, AND, NOR, and NAND logic gates. Scale bar: 50 µm. (b) Circuit diagram and input–output signal response of the NOT gate. (c) Circuit diagrams and corresponding input–output signal characteristics of the NOR, NAND, and AND gates.

## 3 Conclusions

In summary, we have developed an FLA process that enables rapid conversion of metal oxide sol‐gels into functional oxides on both silicon and UTG substrates without requiring additional light‐absorbing layers, through the strategic use of a mullite chuck. FEA simulations demonstrate that the transformation is initiated by direct light absorption within the metal oxides, although this inference awaits more direct experimental verification. The low thermal conductivity of mullite effectively suppresses heat dissipation, allowing temperature to accumulate in the metal oxide layers as the number of light pulses increases, thereby promoting complete gel‐to‐oxide conversion. Simulations further reveal that metal oxides on UTG substrates reach lower temperatures than those on silicon, due to greater heat dissipation into the UTG substrate. The FLA process converts the gels into oxides within tens of seconds, yielding electrical properties comparable to or better than those achieved by CTA and RTA, which require hours and tens of minutes, respectively. The electrical performance of the metal oxide patterns improves with increasing pulse number, attributable to enhanced crystallization. Using FLA‐processed metal oxide patterns, we have successfully fabricated all‐solution‐processed MOTFTs and functional logic gates. This approach reduces annealing time by two orders of magnitude without compromising device performance, demonstrating its strong potential for high‐rate manufacturing of complex integrated circuits. It should be noted that the current study is primarily limited to In_2_O_3_, ITO, and Al_2_O_3_ thin films on silicon and UTG substrates. Nevertheless, the strategy of enabling targeted heat accumulation by selecting low‐thermal‐conductivity chuck materials is expected to be extendable to the annealing of other solution‐processed transparent films. Future work will focus on expanding the library of applicable materials and compatible substrates, as well as developing in‐situ temperature measurement techniques to directly validate the FEA simulation results.

## 4 Experimental Section

### 4.1 Preparation of Metal Oxide Precursor Solutions

All precursor solutions were prepared by dissolving the corresponding metal salts in 2‐Methoxyethanol (2‐MOE, AR grade). The ITO precursor (0.6 m) was formulated by dissolving 6.50 g of indium nitrate hydrate (In(NO_3_)_3_·xH_2_O, 99.999%) and 0.54 g of tin (II) chloride dihydrate (SnCl_2_·2H_2_O, 99.999%) in 40 mL of 2‐MOE. The In_2_O_3_ precursor (0.2 m) was obtained by dissolving 2.41 g of indium nitrate hydrate in 40 mL of 2‐MOE. The Al_2_O_3_ precursor (0.6 M) was prepared by dissolving 9.00 g of aluminum nitrate nonahydrate (Al(NO_3_)_3_·9H_2_O, 99.99%) in 40 mL of 2‐MOE. All chemicals were purchased from Aladdin without further purification. Each solution was stirred for 12 h prior to use.

### 4.2 Preparation of Functionalized Substrates

Ultra‐thin glass (145 µm, SCHOTT AS 87 eco, Shanghai Gous Optics Co., Ltd) and highly doped silicon wafers (500 µm thickness, with a 100 nm thermal oxide layer, Top Vendor Science & Technology Co., Ltd) were used as substrates. The substrates were first treated with oxygen (O_2_) plasma (100 sccm, 180 s) to make their surfaces hydrophilic. They were then transferred to a petri dish, into which 20 µL of Trimethoxy(octyl)silane (TMOS, Aladdin) was added. The dish was heated at 150°C on a hotplate for 10 min to render the substrate surfaces hydrophobic. Subsequently, the TMOS‐coated substrates were irradiated through a photomask for 300 s using a vacuum ultraviolet excimer lamp (π‐Photon, Cygnus Photonics) under a nitrogen atmosphere (99.999% purity) flowing at 15 L min^−1^. The ultraviolet wavelength was 172 nm, and the emission intensity was 10 ± 2 mW cm^−2^. The exposed regions of the substrates became hydrophilic, yielding functionalized substrates with well‐defined wettability contrast patterns. A schematic illustration detailing the fabrication process of these functionalized substrates can be found in Note S20, as well as in previous studies [60, 61].

### 4.3 Fabrication of Metal Oxide Patterns Utilizing the SEDA Process

The SEDA process was carried out in an environmentally controlled chamber (Yipin Instrument Co., Ltd.) maintained at 25°C and 10% relative humidity. Functionalized substrates were immersed in metal oxide precursor solutions and withdrawn at a constant speed of 5 mm s^−1^ using a dip‐coater (SYDC‐1, Sanyan Laboratory Instrument Co., Ltd.). The precursor solutions adhered exclusively to the hydrophilic pattern regions, forming a patterned wet gel film. Subsequent annealing converted these gels into metal oxide patterns.

### 4.4 Different Annealing Processes and Corresponding Annealing Parameters

The wet gel patterns were preheated on a hotplate at 150°C for 3 min prior to annealing. For the conventional thermal annealing process, the hotplate was heated to 500°C, and samples were then placed on it for durations ranging from 20 s to 3 h. For the rapid thermal annealing process, the process consisted of gas purging for 1 min, preheating at 200°C for 1 min, annealing at 500°C for 20 s to 3 min, and cooling to room temperature. For the flash lamp annealing process, the process program was conducted at 1100 V with a pulse width of 10 ms, a pulse interval of 1 s, and varying pulse numbers. Samples were positioned at the center of the chucks, 1 cm from the lamp.

### 4.5 Simulation of Temperature Profiles During the FLA Process

The temperature distribution within the ITO/substrate/chuck stack during the FLA process was simulated using COMSOL Multiphysics. All material parameters employed in the simulation are provided in Note S4. The simulation model incorporates the following geometrical parameters: ITO film thickness of 100 nm, silicon substrate thickness of 500 µm, UTG substrate thickness of 145 µm, and chuck thicknesses of 10 mm (mullite), 20 mm (graphite), and 5 mm (aluminum). The thermal response under light irradiation is governed by the heat conduction equation:

(1) $$\rho C_{p}\frac{\partial T}{\partial t}=\nabla \left(k\nabla T\right)+Q$$

where *T* is the temperature, ρ is the density, *C_p_* is the isobaric specific heat capacity, *t* is the time, *k* is the thermal conductivity, and *Q* is the volumetric heat generation rate. The volumetric heat absorption rate is given by:

(2) $$Q=\alpha I_{x}$$

where α is the absorption coefficient and *I_x_* is the transmitted light intensity at depth *x*. Light attenuation within each material layer follows the Beer–Lambert law [62]:

(3) $$I_{x}=I_{0}\;e^{-\alpha x}$$

where *I*_0_ is the light intensity just inside the absorbing medium, which has already subtracted the reflection loss at that interface. In our simulation, the lamp output incident on the ITO layer is modeled as a wavelength‐independent pulse with an intensity of 1.12 kW cm^−2^. Because light incident on a material layer undergoes multiple reflections at its top and bottom interfaces, multiple absorptions occur within the layer. Equation (3) is therefore corrected to:

(4) $$I_{x}=\frac{I_{0}\left(e^{-\alpha x}+R_{1}e^{-\alpha \left(2L-x\right)}\right)}{1-R_{0}R_{1}e^{-2\alpha L}}$$

where *L* is the total thickness of the current material layer, *R*_0_ is the interfacial reflectance between the current material and the overlying layer, and *R*_1_ is the interfacial reflectance between the current material and the underlying layer. Detailed calculations of the light intensity within a single layer are provided in Note S5. For the multilayer stack studied here, the light emerging from both the overlying and underlying layers acts as incident light on a given layer. Equation (4) is further extended to:

(5) $$I_{x}=\frac{I_{0}\left(e^{-\alpha x}+R_{1}e^{-\alpha \left(2L-x\right)}\right)}{1-R_{0}R_{1}e^{-2\alpha L}}+\frac{I_{1}\left(e^{-\alpha \left(L-x\right)}+R_{0}e^{-\alpha \left(L+x\right)}\right)}{1-R_{0}R_{1}e^{-2\alpha L}}$$

where *I*_1_ is the intensity of the light emerging from the underlying layers.

The boundary conditions incorporate both natural convection and radiation:

(6) $$k\nabla T\;n=h\left(T-T_{amb}\right)+\varepsilon \sigma \left(T^{4}-{T_{amb}}^{4}\right)$$

where ***n*** is the unit normal vector pointing outward from the boundary, *h* is the convective heat transfer coefficient (taken as 15 W m^−2^ K^−1^, a typical value for natural convection in air), *T*_amb_ is the ambient temperature (25°C), *ε* is the surface emissivity (0.95, applicable to a wide range of non‐metallic materials), and σ is the Stefan–Boltzmann constant (‌5.67 × 10^−8^ W m^−2^ K^−4^). In the radiation term, a fixed ambient temperature is employed. This simplification is justified because the chamber wall is large and remains at a constant temperature through thermal equilibrium with the surrounding environment, effectively forming a blackbody enclosure. Furthermore, the sample is sufficiently small that its emitted radiative power is negligible compared to the incident blackbody flux from the wall, and thus does not measurably alter the wall temperature. Approximating the surrounding environment as a blackbody at a constant temperature is an engineering simplification for treating the radiative heat transfer of small samples.

The cumulative heat loss per unit area from a material layer, denoted as *Q_d_*, consists of the contributions from the top and bottom interfaces and is obtained by integrating the heat flux over time:

(7) $$Q_{d}=Q_{d,0}+Q_{d,L}=\int_{t_{1}}^{t_{2}}{\left.-k\frac{\partial T}{\partial x}\right|}_{x=0}dt+\int_{t_{1}}^{t_{2}}{\left.-k\frac{\partial T}{\partial x}\right|}_{x=L}dt$$

where *Q*_*d*,0_ and *Q*_*d*,*L*_ represent the time‑integrated heat fluxes (total heat transfer per unit area) across the top (*x* = 0) and bottom (*x* = *L*) interfaces of the layer, respectively, while *t*_1_ and *t*_2_ denote the start and end times of the heat transfer process.

### 4.6 Device and Metal Oxide Pattern Characterization

Optical images of the metal oxide patterns and devices were captured using an optical microscope (XJ‐52C, PuZhe Optoelectronics). The thickness and root‐mean‐square (RMS) roughness were determined by atomic force microscopy (MultiMode 8, Bruker). The chemical composition was analyzed via X‐ray photoelectron spectroscopy (PHI Quantera II, ULVAC‐PHI). During the analysis, the binding energies were calibrated with reference to the C 1s peak to ensure accuracy. The crystal structure was examined by X‐ray diffraction (D/max‐2550, Rigaku), with Cu Kα radiation generated at 60 kV and 300 mA. The ultraviolet photoelectron spectroscopy measurement was performed on an XPS system (ESCALAB Xi+, Thermo Fisher Scientific) equipped with a He I discharge lamp (21.22 eV) to probe the occupied electronic states near the Fermi level. The Fermi level was calibrated using a gold reference. The optical absorption spectrum was recorded using a UV‑‐vis spectrophotometer (Lambda 950, PerkinElmer). The microstructural characterization was conducted using transmission electron microscopy (JEM‐2100F, JEOL). Aluminum electrodes (∼50 nm thick) were deposited by thermal evaporation and patterned using a combination of photolithography and lift‐off techniques. The electrical properties of the metal oxide patterns, thin‐film transistors, and logic gates were evaluated under ambient conditions using a semiconductor parameter analyzer (B1500A, Keysight Technologies) in conjunction with a probe station (TS2000‐SE, MPI).

## Author Contributions

**Jingwei Zhang**: methodology, validation, investigation. **Wei He**: validation, methodology, investigation, data curation, visualization. **Zetong Li**: writing – original draft, validation, visualization, data curation, methodology. **Guangji Wang**: software, methodology, validation, data curation, investigation. **Zhimin Chai**: writing – review and editing, resources, data curation, methodology, conceptualization, funding acquisition, investigation, validation, formal analysis, supervision, project administration. **Xinchun Lu**: resources, supervision, project administration, funding acquisition.

## Conflicts of Interest

The authors declare no conflict of interest.

## Supporting information




**Supporting File**: advs77200‐sup‐0001‐SuppMat.docx.

## Data Availability

The data that support the findings of this study are available from the corresponding author upon reasonable request.
