# Supplementary material for: Light‐Absorbing‐Layer‐Free Flash Lamp Annealing for High‐Throughput Fabrication of All‐Solution‐Processed Oxide Electronics
Source: Adv Sci (Weinh). 2026 Aug 13:e77200. Online ahead of print. doi: 10.1002/advs.77200 (PMC13472120; doi:10.1002/advs.77200)
Supplement: Supplementary file 1 [file ADVS-9999-e77200-s001.docx]

# Supporting Information

**Light-Absorbing-Layer-Free Flash Lamp Annealing for High-Throughput Fabrication of All-Solution-Processed Oxide Electronics**

Zetong Li,^1,2^ Wei He,^1,2^ Guangji Wang ^1,2^ Jingwei Zhang,^1,2^ Zhimin Chai,^1,2^* and Xinchun Lu^1,2^

^1^ Beijing Key Laboratory of Atomic-Scale Precision Manufacturing and Cross-Scale Device Fabrication, Department of Mechanical Engineering, Tsinghua University, Beijing, China

^2^ State Key Laboratory of Tribology in Advanced Equipment, Tsinghua University, Beijing 100084, China

E-mail: chaizhimin@mail.tsinghua.edu.cn

**Table of contents:**

**Note 1.** Resistivity characterization of ITO patterns via the four-point probe method.

**Note 2.** Benchmarking the resistivity of FLA-processed ITO patterns with literature values.

**Note 3.** Comparison of the resistivity of ITO patterns processed via FLA versus those treated with CTA and RTA.

**Note 4.** Material parameters employed in the simulation of temperature profiles during the FLA process.

**Note 5.** Calculation of transmitted light intensity and light adsorption ratio within a material layer.

**Note 6.** Robustness assessment of model response under parameter uncertainty.

**Note 7.** Refined simulation model incorporating temperature‑dependent input parameters.

**Note 8.** Auxiliary thermal evaluation using temperature‑indicating strips.

**Note 9.** Evolution of thickness, surface roughness, and atomic bonding states in ITO patterns with pulse number.

**Note 10.** Evolution of thickness, surface roughness, dielectric constant, and atomic bonding states in Al_2_O_3_ patterns with pulse number.

**Note 11.** Evolution of dielectric and insulating properties of FLA-processed Al_2_O_3_ patterns with pulse number.

**Note 12.** Evolution of thickness, surface roughness, atomic bonding states, and crystal structure in In_2_O_3_ patterns with pulse number.

**Note 13.** Structural layout and fabrication process flow of the MOTFTs.

**Note 14.** Transfer characteristic of MOTFTs under 20-pulse annealing.

**Note 15.** Comparison of the electrical performance of MOTFTs in this work and in the literature.

**Note 16.** Stability test of the electrical performance of MOTFTs fabricated via FLA.

**Note 17.** Comprehensive characterization of key electronic and optical properties: work function (*Φ*), energy difference between the valence band maximum and the Fermi level (*E*_VBM-F_), and optical bandgap (*E*_g_) for both In_2_O_3_ and ITO.

**Note 18.** Long-term stability of all‑solution‑processed logic circuits fabricated via FLA.

**Note 19.** Dynamic response of logic gate circuits to square-wave input signals.

**Note 20.** Schematic illustration of the fabrication of functionalized substrates.

**Note 1.** **Resistivity characterization of ITO patterns via the four-point probe method.**

The resistivity of assembled ITO patterns is characterized by the four-probe method. To probe the ITO patterns, four aluminum electrodes are fabricated using a combination of photolithography, thermal evaporation and lift-off processes. The optical micrograph of the four-probe test structure is shown in Figure S1a. During the four-probe measurement, a variable current is applied between electrodes 1 and 2, and a voltage is measured between electrodes 3 and 4. The measured voltage as a function of the applied current is plotted in Figure S1b. The slope of the voltage-current curve is the resistance of the ITO pattern between electrodes 3 and 4. Using the Ohm's law, the resistivity of the ITO pattern can be calculated as

$\rho=\frac{t w}{l}\frac{V}{I}$	(S1)

where *t* and *w* are the thickness and width of the ITO pattern, *l* is the length of the ITO pattern between electrodes 3 and 4, *V* and *I* are the voltage and current. The width, length and thickness of the ITO pattern are 40 μm, 100 μm and 32.7 nm, respectively. The spacing between two adjacent aluminum electrodes is 20 μm. According to Equation S1, the resistivity of the ITO pattern in Figure S1 is calculated to be 4.3 × 10^-3^ Ω cm.

**Figure S1.** (a) Optical image of resistivity testing for ITO using the four-point probe method. (b) Measured voltage between electrodes 3 and 4 as a function of the applied current.

**Note 2.** **Benchmarking the resistivity of FLA-processed ITO patterns with literature values.**

Resistivity values of 4.3 × 10^-3^ and 2.2 × 10^-3^ Ω cm are obtained for FLA-processed ITO patterns subjected to 20 and 200 pulses, respectively. These values are either comparable to or even lower than those reported for ITO films fabricated and annealed by other methods (Table S1).

**Table S1.** Benchmarking the resistivity of FLA-processed ITO patterns with literature values.

| **Fabrication**  **method** | **Annealing method** | **Annealing time** | **Thickness**  **(nm)** | **Resistivity**  **(Ω cm)** | **Ref.** |
| --- | --- | --- | --- | --- | --- |
| Sputtering | CTA | 3600 s at 250 ℃ | 178 | 5 × 10^-4^ | [1] |
|  | FLA | ＜1 s | 178 | 9.5 × 10^-4^ | [1] |
| Electron beam deposition | RTA | Total 4440 s, at 550 ℃ for 600 s | 60 | 1.2 × 10^-3^ | [2] |
|  | FLA | 20 s | 60 | 6.6 × 10^-3^ | [2] |
| Inkjet printing | FLA | ~130 s | 70 | 3.1 × 10^-3^ | [3] |
| Dip-coating of precursor solution | CTA | 3600 s at 500 ℃ | 105 | 7.7 × 10^-3^ | [4] |
|  | CTA | 3600 s at 500 ℃ | 125 | 4.9 × 10^-3^ | [4] |
|  | CTA | 3600 s at 500 ℃ | 145 | 2.9 × 10^-3^ | [4] |
|  | RTA | Total time＞360 s, at 550 ℃ for 360 s | 320 | 3.6 × 10^-3^ | [4] |
| SEDA | CTA | 3600 s at 400 ℃ | 24.4 | 1.9 × 10^-3^ | [5] |
| SEDA | FLA | ~20 s | 32.7 | 4.3 × 10^-3^ | This work |
|  | FLA | ~200 s | 29.5 | 2.2 × 10^-3^ | This work |

**Note 3.** **Comparison of the resistivity of ITO patterns processed via FLA versus those treated with CTA and RTA.**

Figure S2a shows the resistivity of ITO patterns fabricated on silicon substrates and subsequently annealed on a mullite chuck using varying numbers of light pulses. With only 10 pulses (~ 10 s), the resistivity drops to 1.0 × 10^-2^ Ω cm. Increasing the pulse count to 20 (~ 20 s) reduces the resistivity further to 4.3 × 10^-3^ Ω cm, while 200 pulses (~ 200 s) lower it to 2.2 × 10^-3^ Ω cm.

For comparison, when the ITO patterns are annealed via conventional thermal annealing (CTA) at 500 ^o^C for 20 s, the resistivity is 2.6 × 10^-2^ Ω cm (Figure S2b), which is approximately 6 times higher than that obtained with 20 s of FLA. Extending the CTA duration to 1 h lowers the resistivity to 6.3 × 10^-3^ Ω cm, a value still higher than that achieved with 20 s of FLA. Prolonging the CTA time to 3 h yields only a slight reduction to 4.5 × 10^-3^ Ω cm.

In the rapid thermal annealing (RTA) process, the total processing time includes gas stabilization (60 s), heating (60 s), holding (20 s to 3 min), and cooling (560 s). Annealing at 500 ^o^C with a 20 s hold (total time ~12 min) results in a resistivity of 8.7 × 10^-3^ Ω cm. Increasing the holding time to 3 min (total ~14 min) lowers the resistivity to 4.2 × 10^-3^ Ω cm. While higher annealing temperatures generally lower resistivity, ITO patterns annealed at 800 ^o^C with a 20 s hold still show a higher resistivity (6.0 × 10^-3^ Ω cm) than those treated with 20 s of FLA.

**Figure S2.** (a) The resistivity of ITO patterns processed with FLA at varying number of pulses. (b) The resistivity of ITO patterns processed with CTA at varying annealing time. (c) The resistivity of ITO patterns processed with RTA at varying annealing time.

**Note 4.** **Material parameters employed in the simulation of temperature profiles during the FLA process.**

The material parameters employed in the simulation are listed in Table S2, including density (*ρ*), isobaric specific heat capacity (*C_p_*), thermal conductivity (*k*), and thickness (*L*).

**Table S2.** Parameters of the materials used in the simulation.

|  | **Graphite**^[6]^ | **Aluminum^[7]^** | **Mullite**^[8]^ | **UTG**^[9]^ | **Silicon**^[10]^ | **ITO**^[11]^ |
| --- | --- | --- | --- | --- | --- | --- |
| ***ρ* (kg m^-3^)** | 1850 | 2698 | 1200 | 2460 | 2329 | 7120 |
| ***k* (****W m^-1^ K^-1^)** | 140 | 234 | 0.4 | 1.2 | 148 | 5.3 |
| ***C_p_* (J kg^-1^ K^-1^)** | 710 | 899 | 880 | 870 | 705 | 350 |
| ***L* (mm)** | 20 | 5 | 10 | 0.145 | 0.5 | 0.0001 |

The spectrum of the xenon lamp used in this work is shown in Figure S3a. The emission covers the ultraviolet to near‑infrared range, with the highest intensity in the visible region. Figure S3b presents the measured reflectance spectra for silicon, mullite, and UTG, together with literature values for ITO,^[12]^ aluminum,^[13]^ and graphite.^[14]^ Figure S3c displays the optical absorption coefficient ($\alpha$) derived from the extinction coefficients ($\kappa$) of ITO,^[12]^ graphite,^[14]^ aluminum,^[15]^ mullite,^[16]^ and silicon^[17]^ using the following relation:^[18]^

$\alpha=\frac{4\pi\kappa}{\lambda}$	(S2)

where *λ* is the wavelength. For UTG, the absorption coefficient is calculated from its measured transmission and reflection spectra via:^[18-19]^

$\alpha=-\frac{1}{L}\left\{ \frac{T^{2}-\left( 1-R \right)^{2}+\left\{ \left[ T^{2}-\left( 1-R \right)^{2} \right]^{2}+4T^{2} \right\}^{1/2}}{2T} \right\}$	(S3)

where *T* and *R* denote the transmittance and reflectance of UTG, respectively.

**Figure S3.** (a) Spectrum of the xenon lamp used in this study. (b) Reflectance of the materials considered. (c) Absorption coefficients of the materials.

Wavelength‑dependent optical parameters pose considerable computational challenges in finite‑element photothermal simulations. In radiative‑heat‑transfer and laser‑processing studies, it is common practice to use wavelength‑independent equivalent parameters.^[20-23]^ The equivalent reflectance is obtained as a light‑intensity‑weighted average:^[24]^

$\tilde{R}=\frac{\int I_{0}\left( \lambda\right)R(\lambda)d\lambda}{\int I_{0}\left( \lambda\right)d\lambda}$	(S4)

where $I_{0}\left( \lambda\right)$ is the spectral intensity of the incident light.

The equivalent absorption coefficient ($\tilde{\alpha}$) is derived from the spectral absorption coefficient ($\alpha\left( \lambda\right)$), reflectance ($R(\lambda)$), and material depth ($x$) according to the relation:^[23]^

$\tilde{\alpha}(x)e^{-\tilde{\alpha}(x)x}=\frac{\int(1-R(\lambda))I_{0}\left( \lambda\right)\alpha\left( \lambda\right)\text{e}^{-\alpha\left( \lambda\right)x}d\lambda}{\int(1-R(\lambda))I_{0}\left( \lambda\right)d\lambda}$	(S5)

$\tilde{\alpha}=\frac{\sum_{i} \tilde{\alpha}(x_{i})\text{e}^{-\tilde{\alpha}(x_{i})x_{i}}}{\sum_{i} \text{e}^{-\tilde{\alpha}(x_{i})x_{i}}}$	(S6)

**Table S3.** Optical parameters used in the simulation.

|  | **Graphite** | **Aluminum** | **Mullite** | **UTG** | **Silicon** | **ITO** |
| --- | --- | --- | --- | --- | --- | --- |
| $\tilde{\boldsymbol{\alpha}}$ **(*m*^-1^)** | 25656888 | 122190804 | 7488 | 375 | 1024975 | 544216 |
| $\tilde{\boldsymbol{R}}$ **(%)** | 22 | 79 | 67 | 9 | 42 | 13 |

For normal incidence from material 1 to material 2, the interfacial reflectance ($R_{12}$) is given by:^[25]^

$R_{12}=\left( \frac{n_{2}-n_{1}}{n_{2}+n_{1}} \right)^{2}=\left( \frac{\sqrt{R_{2}}-\sqrt{R_{1}}}{1-\sqrt{R_{1}R_{2}}} \right)^{2}$	(S7)

where $n_{1}$, $R_{1}$ and $n_{2}$, $R_{2}$ are the refractive indices and reflectance values (relative to air) of the two materials, respectively.

**Table S4.** Optical parameters used in the simulation.

|  | **ITO/Silicon** | **ITO/UTG** | **UTG/Mullite** | **Silicon/Mullite** | **Silicon/Aluminum** | **Silicon/Graphite** |
| --- | --- | --- | --- | --- | --- | --- |
| ***R*_12_ (%)** | 14.1 | 0.5 | 47.2 | 13.2 | 32.4 | 6.6 |

**Note 5.** **Calculation of** **transmitted light intensity and light adsorption ratio within a material layer.**

When light of total intensity $I_{total}$ is incident on a material layer of thickness *L* (Figure S4), it is first reflected at interface 0. The intensity transmitted into the material ($I_{0}$) and the reflected portion ($I_{r}$) are respectively denoted as

$I_{0}=(1-R_{0}) I_{\text{total}}$	(S8)

$I_{r}=R_{0}I_{\text{total}}$	(S9)

The transmitted light then undergoes multiple reflections between interface 0 and interface 1, while exiting from both interfaces. According to the Beer‑Lambert law,^[26]^ the intensity reaching interface 1 after traversing the layer is

$I_{\text{t}}=I_{0}\text{e}^{-\alpha L}$	(S10)

where $\text{α}$ is the absorption coefficient of the material. The first emergent beam at interface 1 ($I_{t1,1}$) and the first reflected beam at that interface ($I_{r1,1}$) are

$I_{\text{t1,1}}=\left( 1-R_{1} \right)I_{\text{t}}=\left( 1-R_{1} \right)I_{0}\text{e}^{-\alpha L}$	(S11)

$I_{\text{r1,1}}=R_{1}I_{t}=R_{1}I_{0}\text{e}^{-\alpha L}$	(S12)

After propagating back through the layer, the first emergent beam at interface 0 ($I_{t0,1}$) and the first reflected beam at that interface ($I_{r0,1}$) are

$I_{\text{t0,1}}=\left( 1-R_{0} \right)I_{\text{r1,1}}\text{e}^{-\alpha L}=\left( 1-R_{0} \right)R_{1}I_{0}\text{e}^{-2\alpha L}$	(S13)

$I_{\text{r0,1}}=R_{0}I_{\text{r1,1}}\text{e}^{-\alpha L}=R_{0}R_{1}I_{0}\text{e}^{-2\alpha L}$	(S14)

Similarly, the second emergent beams at interface 1 ($I_{t1,2}$) and interface 0 ($I_{t0,2}$) are

$I_{\text{t1,2}}=\left( 1-R_{1} \right)I_{\text{r0,1}}\text{e}^{-\alpha L}=\left( 1-R_{1} \right)R_{0}R_{1}I_{0}\text{e}^{-3\alpha L}$	(S15)

$I_{\text{t0,2}}=\left( 1-R_{0} \right)I_{\text{r1,2}}\text{e}^{-\alpha L}=\left( 1-R_{0} \right)R_{0}R_{1}^{2}I_{0}\text{e}^{-4\alpha L}$	(S16)

Summing over all reflections gives the total emergent intensities at interface 1 ($I_{t1}$) and interface 0 ($I_{t0}$):

$I_{t1}=I_{\text{t1,1}}+I_{\text{t1,2}}+\ldots=(1-R_{1})I_{0}\text{e}^{-\alpha L}\sum_{i=0}^{n} \left( R_{0}R_{1}\text{e}^{-2\alpha L} \right)^{n}=\frac{(1-R_{1})I_{0}\text{e}^{-\alpha L}}{1-R_{0}R_{1}\text{e}^{-2\alpha L}}$	(S17)

$I_{t0}=I_{t0,1}+I_{t0,2}+\ldots=\left( 1-R_{0} \right)R_{1}I_{0}\text{e}^{-2\alpha L}\sum_{i=0}^{n} \left( R_{0}R_{1}\text{e}^{-2\alpha L} \right)^{n}=\frac{\left( 1-R_{0} \right)R_{1}I_{0}\text{e}^{-2\alpha L}}{1-R_{0}R_{1}\text{e}^{-2\alpha L}}$	(S18)

The total light intensity at a position *x* inside the layer is

$I_{x}=I_{0}\text{e}^{-\alpha x}+I_{\text{r1,1}}\text{e}^{-\alpha(L-x)}+I_{\text{r0,1}}\text{e}^{-\alpha x}+\ldots=\frac{I_{0}(\text{e}^{-\alpha x}+R_{1}\text{e}^{-\alpha(2L-x)})}{1-R_{0}R_{1}\text{e}^{-2\alpha L}}$	(S19)

**Figure S4.** Schematic of multiple reflections at the top and bottom interfaces of a material layer during light propagation.

In practice, the layer may also receive light entering from interface 1 with intensity $I_{1}$ (Figure S5). When both incident beams are present, the total emergent intensities and the internal intensity become the sum of contributions from each incident beam:

$I_{t1}=\frac{(1-R_{1})I_{0}\text{e}^{-\alpha L}}{1-R_{0}R_{1}\text{e}^{-2\alpha L}}+\frac{\left( 1-R_{1} \right)R_{0}I_{1}\text{e}^{-2\alpha L}}{1-R_{0}R_{1}\text{e}^{-2\alpha L}}$	(S20)

$I_{t0}=\frac{\left( 1-R_{0} \right)R_{1}I_{0}\text{e}^{-2\alpha L}}{1-R_{0}R_{1}\text{e}^{-2\alpha L}}+\frac{(1-R_{0})I_{1}\text{e}^{-\alpha L}}{1-R_{0}R_{1}\text{e}^{-2\alpha L}}$	(S21)

$I_{x}=\frac{I_{0}(e^{-\alpha x}+R_{1}\text{e}^{-\alpha(2L-x)})}{1-R_{0}R_{1}\text{e}^{-2\alpha L}}+\frac{I_{1}(e^{-\alpha(L-x)}+R_{0}\text{e}^{-\alpha(L+x)})}{1-R_{0}R_{1}\text{e}^{-2\alpha L}}$	(S22)

**Figure S5.** Schematic of light entering the layer from both the top and bottom interfaces.

The light absorbed by the layer is the difference between the total incident intensity and the total exiting intensity. Thus, the fraction of the total incident light absorbed by the layer ($A_{1}$) is:

$A_{1}=\frac{\left( I_{0}+I_{1} \right)-(I_{\text{t0}}+I_{\text{t1}})}{I_{\text{total}}}$	(S23)

**Note 6.** **Robustness assessment of model response under parameter uncertainty.**

To assess whether the temperature evolution and energy partitioning depend on the input parameters used in the finite element simulations, we performed a perturbation-based robustness analysis using the ITO/silicon/mullite stack as a test structure. The analysis considers six categories of input parameters—thermal conductivity, density, specific heat capacity, optical absorption coefficient, interface reflectance, convective heat-transfer coefficient, and surface emissivity—totaling 17 independent parameters. These parameters are assigned perturbation ranges that encompass both measurement errors and sample-to-sample variations. The thermophysical parameters are assumed to follow a normal distribution, with the fixed values used in this study serving as the mean values and a standard deviation of ±30%, while the reflectance and emissivity values are assumed to follow a uniform distribution. The range of each parameter, along with the values for the fixed input parameters used in this study, is listed in Table S5. A Latin hypercube sampling strategy is employed to generate 200 independent random parameter sets, and the finite element simulation is repeated for each set. After 20 FLA light pulses, the following outputs are extracted: the temperatures at all interfaces, the temperature difference between the top and bottom surfaces of the ITO layer, the optical energy absorbed, the energy dissipated, and the net energy retained within the ITO layer.

**Table S5.** Parameters utilized in the perturbation-based robustness analysis.

| **Parameters** | **Distribution** | **Range** | **Fixed input parameters** |
| --- | --- | --- | --- |
| $\boldsymbol{k}_{\mathbf{ITO}}$ **(W m^-1^ K^-1^)** | Normal | 5.3 ± 1.59 | 5.3 |
| $\boldsymbol{k}_{\mathbf{Si}}$ **(W m^-1^ K^-1^)** | Normal | 148 ± 44.4 | 148 |
| $\boldsymbol{k}_{\mathbf{mullite}}$ **(W m^-1^ K^-1^)** | Normal | 0.4 ± 0.12 | 0.4 |
| $\boldsymbol{C}_{\boldsymbol{p}\mathbf{,}\mathbf{ITO}}$ **(J kg^-1^ K^-1^)** | Normal | 350 ± 105 | 350 |
| $\boldsymbol{C}_{\boldsymbol{p}\mathbf{,}\mathbf{Si}}$ **(J kg^-1^ K^-1^)** | Normal | 705 ± 211.5 | 705 |
| $\boldsymbol{C}_{\boldsymbol{p}\mathbf{,}\mathbf{mullite}}$ **(J kg^-1^ K^-1^)** | Normal | 880 ± 264 | 880 |
| $\boldsymbol{\rho}_{\mathbf{ITO}}$ **(kg m^-3^)** | Normal | 7120 ± 2136 | 7120 |
| $\boldsymbol{\rho}_{\mathbf{Si}}$ **(kg m^-3^)** | Normal | 2329 ± 698.7 | 2329 |
| $\boldsymbol{\rho}_{\mathbf{mullite}}$ **(kg m^-3^)** | Normal | 1200 ± 360 | 1200 |
| $\boldsymbol{\alpha}_{\text{ITO}}$ **(m^-1^)** | Normal | 544216 ± 163264 | 544216 |
| $\boldsymbol{\alpha}_{\text{Si}}$ **(m^-1^)** | Normal | 1024975 ± 307492 | 1024975 |
| $\boldsymbol{h}_{\mathbf{air}\mathbf{/}\mathbf{ITO}}$ **(W m^-2^ K^-1^)** | Normal | 15 ± 4.5 | 15 |
| $\boldsymbol{h}_{\mathbf{mullite}\mathbf{/}\mathbf{air}}$ **(W m^-2^ K^-1^)** | Normal | 15 ± 4.5 | 15 |
| $\boldsymbol{R}_{\mathbf{air}\mathbf{/}\mathbf{ITO}}$ | Uniform | [0.078, 0.182] | 0.13 |
| $\boldsymbol{R}_{\mathbf{ITO}\mathbf{/}\mathbf{S}\mathbf{i}}$ | Uniform | [0.085, 0.197] | 0.141 |
| $\boldsymbol{\varepsilon}_{\mathbf{air}\mathbf{/}\mathbf{ITO}}$ | Uniform | [0.8, 1] | 0.95 |
| $\boldsymbol{\varepsilon}_{\mathbf{mullite}\mathbf{/}\mathbf{air}}$ | Uniform | [0.8, 1] | 0.95 |

As shown in Figure S6, all the output parameters exhibit relatively large absolute fluctuations, which are attributable to the deliberately wide perturbation range imposed on the input parameters. Nevertheless, the mean values of each output parameter remain consistent with the values obtained using the fixed input parameters in this study. For example, the mean temperature at the air/ITO interface is 538.91 ℃, compared to 530.58 ℃ when using the fixed input parameters. Similarly, the mean value of the temperature difference Δ*T* across the ITO layer is 5.85 × 10^-3^ °C, which is also very close to the value of 5.07 × 10^-3^ °C obtained using the fixed input parameters.

Although deviations in the output parameters are observed, the temperature difference between the top and bottom surfaces of the ITO layer and the net energy retained within the ITO layer remain positive across the entire range of input parameter variations. These results further substantiate the main conclusion that oxide conversion is initiated by direct light absorption within the metal oxide layer, while also demonstrating the robustness of our simulation method.

**Figure S6.** Output parameters extracted after 20 pulses. (a-d) Temperatures at the (a) air/ITO, (b) ITO/Si, (c) Si/mullite, and (d) mullite/air interfaces. (e) Temperature difference between the air/ITO and ITO/Si interfaces. (f) Absorbed energy in the ITO layer. (g) Dissipated energy from the ITO layer. (h) Net energy retained in the ITO layer.

To further quantify the influence of each thermophysical parameter on the core conclusions of this work, we performed a series of controlled FEA simulations: for each individual parameter, we varied its value within the predefined range listed in Table S5, while keeping all other thermophysical parameters fixed. The resulting simulated temperature at the air/ITO interface, as well as the temperature difference between the air/ITO and ITO/silicon interfaces, are systematically summarized in Figure S7.

(1) Thermal conductivity: For any component in the ITO/Si/mullite stack, increasing its thermal conductivity (*k*_ITO_, *k*_Si_, or *k*_mullite_) leads to a lower peak temperature at the air/ITO interface, which arises from enhanced heat dissipation across the entire stack. Due to the low thickness of the ITO film (100 nm), with the increase of its thermal conductivity, the temperature decrease at the air/ITO interface remains marginal. As *k*_ITO_ increases, the temperature difference between the air/ITO and ITO/silicon interfaces decreases, driven by accelerated heat transport within the thin ITO layer. In contrast, varying the thermal conductivity of the silicon substrate or mullite chuck exerts negligible influence on the interfacial temperature difference.

(2) Specific heat capacity: For any material in the stack, a higher specific heat capacity (*C_p_*_,ITO_, *C_p_*_,Si_, or *C_p_*_,mullite_) requires more absorbed energy to achieve the same temperature rise, which consequently reduces the peak temperature at the air/ITO interface. Again, owing to the ultra-thin nature of the ITO film, increasing *C_p_*_,ITO_ only induces a minor decrease in the air/ITO interface temperature.

(3) Optical absorption coefficient: Increasing the absorption coefficient of either *α*_ITO_ or *α*_Si_ enhances the total light energy harvested by the stack, leading to a slight rise in the air/ITO interface temperature. Since heat propagates downward and optical absorption is concentrated near the top surface of each layer, elevating *α*_ITO_ causes a more pronounced temperature increase at the top surface of the ITO film than at its bottom surface, which widens the temperature difference across the ITO layer.

(4) Interfacial reflectance: Increasing the reflectance at the air/ITO (*R*_air/ITO_) or ITO/Si (*R*_ITO/Si_) interface redirects more incident light out of the stack, reducing the net energy absorbed by the ITO film and thus lowering the peak temperature at the air/ITO interface. As *R*_air/ITO_ rises, the temperature at the ITO/silicon interface increases, narrowing the temperature difference between the air/ITO and ITO/silicon interfaces. Conversely, increasing *R*_ITO/Si_ elevates the air/ITO interface temperature, resulting in a larger temperature difference across the ITO layer.

(5) Convective heat transfer coefficient and surface emissivity‌: Increasing the convective heat transfer coefficient (*h*_air/ITO_) and surface emissivity (*ε*_air/ITO_) of the exposed surfaces promotes more efficient heat dissipation to the ambient environment, which consistently reduces the peak temperature at the air/ITO interface.

**Figure S7.** Influence of each thermophysical parameter variation on the simulated air/ITO interface temperature (*T*_air/ITO_) and the temperature difference between the air/ITO and ITO/silicon interfaces (△*T*).

**Note 7.** **Refined simulation model incorporating temperature‑dependent input parameters.**

To evaluate the influence of temperature-dependent material properties, a refined model is constructed based on the ITO/silicon/mullite structure, in which the key input parameters are expressed as functions of temperature. The thermal conductivities of different materials are described by power‑law or linear correlations derived from experimental data:^[11, 27-31]^

$k_{\mathrm{ITO}}\left( T \right)=5.3\times\left( \frac{300}{T} \right)^{0.55}$	(S24)

$k_{\mathrm{Si}}\left( T \right)=148\times\left( \frac{300}{T} \right)^{1.3}$	(S25)

$k_{\mathrm{Mullite}}\left( T \right)=0.40+5.8\times{10}^{-4}\left( T-300 \right)$	(S26)

where $k_{\mathrm{ITO}}$, $k_{\mathrm{Si}}$, and $k_{\mathrm{Mullite}}$ denote the temperature-dependent thermal conductivities of ITO, silicon, and mullite, respectively, and *T* is the absolute temperature in Kelvin. The specific heat capacity is represented by a Shomate‑type equation:^[32]^

$\text{C}_{\text{p}}\left( T \right)=A+B\bar{t}+C\bar{t}^{2}+D\bar{t}^{3}+\frac{E}{\bar{t}^{2}}$	(S27)

where $\bar{t}=\frac{T}{1000}$. The coefficients A, B, C, D, and E are constants determined by fitting to the NIST-JANAF thermochemical data (Table S6). The temperature‑dependent density is obtained from the volumetric thermal expansion coefficient:

$\rho\left( T \right)=\frac{\rho}{1+a_{V}\left( T-300 \right)}$	(S28)

where $a_{V}$ is the volumetric thermal expansion coefficient.

For the optical absorption coefficient and the interfacial reflectance, no universal temperature‑dependent expressions are available. For simplicity, a linear temperature dependence is adopted:

$\alpha\left( T \right)=\alpha_{0}+\alpha_{T}\left( T-T_{\text{ref}} \right)$	(S29)

$R\left( T \right)=R_{0}+R_{T}\left( T-T_{\text{ref}} \right)$	(S30)

where *T*_ref_ = 298.15 K, $\alpha_{0}$ and $R_{0}$ are the optical absorption coefficient and interfacial reflectance at 298.15 K, and $\alpha_{T}$ and $R_{T}$ are the temperature‑dependent coefficients for the optical absorption coefficient and interfacial reflectance, respectively. The optical absorption coefficient and interfacial reflectance are estimated by fitting to literature data (Table S6).

**Table S6.** Temperature-dependent input parameters used in the refined model.

|  | **A** | **B** | **C** | **D** | **E** | $a_{V}$ (K^-1^) | $\alpha_{T}$(m^-1^ K^-1^) | $R_{T}$ (K^-1^) |
| --- | --- | --- | --- | --- | --- | --- | --- | --- |
| **ITO** | 245.5 | 439.3 | -229.4 | 0 | -2.31 | 1.9 × 10^−5[33]^ | 27.67^[34]^ |  |
| **Silicon** | 812.4 | 138.8 | -2.951 | 1.499 | -12.61 | 7.7 × 10^−6[35]^ | 5.15^[36-37]^ |  |
| **Mullite** | 450.1 | 1216 | -549.6 | 0 | -0.0543 | 1.6 × 10^−5[38]^ |  |  |
| **Air/ITO** |  |  |  |  |  |  |  | 5.6 × 10^−5[34]^ |
| **ITO/Silicon** |  |  |  |  |  |  |  | 4.7 × 10^−5[36]^ |

All temperature‑dependent parameters are directly incorporated into the heat source terms and the transient heat‑conduction partial differential equation in the simulation model. The simulation results are presented in Figure S8. Compared with the original model, the interface temperatures at the air/ITO, ITO/Si, and Si/mullite interfaces all decrease in the temperature-dependent model, with the air/ITO interface temperature dropping by approximately 33.4 °C. This reduction is primarily attributable to the increase in specific heat capacity with temperature, which necessitates more energy to achieve the same temperature rise. The elevated temperature at the mullite/air interface arises from the increasing thermal conductivity of mullite with temperature. Meanwhile, the temperature difference across the ITO layer increases to 8.82 × 10^-3^ °C, representing an increase of 3.75 × 10^-3^ °C relative to the value calculated using the original model, mainly owing to the degradation of the thermal conductivity of ITO with temperature. Although the temperature-dependent absorption coefficient enhances the optical energy absorbed by the ITO layer, and the reduced thermal conductivity of Si retains more net energy within the ITO, the ITO temperature still decreases owing to the increase in its specific heat capacity.

Although deviations in the output parameters are observed between the refined and original simulation models, the pronounced temperature difference across the ITO layer and the direction of heat transfer remain qualitatively unchanged. This confirms that the core conclusion derived from the original model with temperature‑independent parameters remains robust.

**Figure S8.** Comparison of output parameters calculated using the original and refined models. (a-d) Simulated temperature evolution at air/ITO, ITO/Si, Si/mullite, and mullite/air interfaces. (e) Temperature difference between the air/ITO and ITO/Si interfaces. (f) Absorbed energy in the ITO layer. (g) Dissipated energy from the ITO layer. (h) Net energy retained in the ITO layer.

**Note 8. Auxiliary thermal evaluation using temperature‑indicating strips.**

A qualitative complementary assessment is conducted using temperature‑indicating strips. When the strip is placed directly on the ITO surface (strip/ITO/Si/mullite), it is destroyed after only two FLA pulses owing to its strong absorption of incident light (Figure S9a). The configuration is therefore modified by attaching the strip to the backside of the silicon wafer (ITO/Si/strip/mullite), where it is shielded from direct illumination by the ITO/Si stack and responds exclusively to conductive heat transfer, enabling temperature readout (Figure S9b). It should be noted that the presence of the strip may alter the heat conduction between the silicon and the mullite chuck. Therefore, the measured temperatures are not exact temperature at the Si/mullite interface but are sufficient as a reference. The results clearly indicate that the maximum temperature recorded by the strip increases with the number of pulses (Figure S9c), thus providing direct experimental evidence for the heat accumulation effect.

**Figure S9.** Temperature measurement using temperature‑indicating strips. (a) Strip placed directly on the ITO surface (strip/ITO/Si/mullite). (b) Strip attached to the backside of the silicon wafer (ITO/Si/strip/mullite), flipped to the front side for readout. (c) Maximum temperature recorded by the strip versus pulse number.

**Note 9.** **Evolution of thickness, surface roughness, and atomic bonding states in ITO patterns with pulse number.**

The thickness of ITO patterns (40 μm × 100 μm) is measured using atomic force microscopy (AFM). A representative AFM topography image (scan area 90 μm × 90 μm) is shown in Figure S10a. The volume of each pattern is obtained by numerical integration of the height data in MATLAB, and the average thickness is then calculated by dividing the volume by the pattern area. Figure S10b plots the average thickness of ITO patterns on silicon and UTG substrates as a function of the number of pulses.

**Figure S10.** (a) AFM image of an ITO pattern. Scale bar: 10 μm. (b) Average thickness of ITO patterns on silicon and UTG substrates versus pulse number.

AFM images of ITO films on silicon and UTG substrates after different numbers of pulses are presented in Figure S11. The root‑mean‑square (RMS) roughness (*R*_q_) extracted from these images decreases as the pulse number increases.

**Figure S11.** AFM images of ITO films on silicon and UTG substrates after different numbers of pulses.

X-ray photoelectron spectroscopy (XPS) is used to examine the composition and bonding states of ITO films processed with varying pulse numbers on silicon (Figure S12a) and UTG (Figure S12b) substrates. The O 1s spectra are deconvoluted into three peaks which are located at 529.9, 531.5, and 532.4 eV, representing lattice oxygen atoms in M-O-M bonds, oxygen vacancies (V_O_), and oxygen atoms in hydroxide (M-OH) groups, respectively. The relative proportions of these oxygen states are summarized in Figure S12c. With increasing pulse number, the fraction of M-O-M bonds rises systematically on both silicon and UTG substrates.

**Figure S12.** (a) XPS O1s spectra of ITO films on silicon substrates after different numbers of pulses. (b) XPS O1s spectra of ITO films on UTG substrates after different numbers of pulses. (c) Relative contributions of M-O-M bonds, oxygen vacancies, and M-OH groups as a function of pulse number.

**Note 10.** **Evolution of thickness, surface roughness, dielectric constant, and atomic bonding states in Al_2_O_3_ patterns with pulse number.**

**Figure S13.** (a) AFM image of an Al_2_O_3_ pattern (70 μm × 70 μm). Scale bar: 10 μm. (b) Average thickness of Al_2_O_3_ patterns on silicon and UTG substrates versus pulse number.

**Figure S14.** AFM images of Al_2_O_3_ films on silicon and UTG substrates after different numbers of pulses.

Figure S15 shows the variation of the dielectric constant of Al_2_O_3_ with the number of pulses. The dielectric constant of Al_2_O_3_ on UTG substrates is higher than that on silicon substrates, which may be attributed to a higher concentration of M-OH bonds.

**Figure S15.** Variation of the dielectric constant of Al_2_O_3_ with pulse numbers on silicon and UTG substrates.

XPS is employed to investigate the composition and bonding states of Al_2_O_3_ films processed with different pulse numbers on silicon (Figure S16a) and UTG (Figure S16b) substrates. The O 1s spectra are deconvoluted into two characteristic peaks at 531.1 eV and 532.4 eV, corresponding to lattice oxygen in M-O-M bonds and oxygen in hydroxyl groups (M-OH), respectively. The relative proportions of these oxygen states are plotted in Figure S16c. With increasing pulse count, the proportion of M-O-M bonds rises steadily in Al_2_O_3_ films on both substrates. At 200 pulses, Al_2_O_3_ on silicon exhibits 89.8% M-O-M bonds and 10.2% M-OH bonds, indicating a high degree of oxide conversion. In contrast, Al_2_O_3_ on UTG shows 67.0% M-O-M bonds and 33.0% M-OH bonds, reflecting a comparatively lower conversion extent.

**Figure S16.** (a) XPS O1s spectra of Al_2_O_3_ films on silicon substrates after different numbers of pulses. (b) XPS O1s spectra of Al_2_O_3_ films on UTG substrates after different numbers of pulses. (c) Relative contributions of M-O-M bonds and M-OH groups as a function of pulse number.

**Note 11.** **Evolution of dielectric and insulating properties of FLA-processed Al2O3 patterns with pulse number.**

As a supplementary explanation to the data in Figures 3e and 3f, Figure S17 presents a more detailed evolution of the areal capacitance (at 1 kHz) and leakage current density (at 1 MV cm^-2^) of FLA‑processed Al_2_O_3_ patterns with pulse number. For each annealing condition, at least 10 individual Al_2_O_3_ patterns are measured.

**Figure S17.** (a) Areal capacitance of Al_2_O_3_ patterns fabricated on silicon and UTG substrates as a function of pulse number. The areal capacitance values are measured at a frequency of 1 kHz. (b) Current density of Al_2_O_3_ patterns fabricated on silicon and UTG substrates as a function of pulse number. The current density values are measured at an electric field strength of 1 MV cm^-1^.

**Note 12.** **Evolution of thickness, surface roughness, atomic bonding states, and crystal structure in In_2_O_3_ patterns with pulse number.**

**Figure S18.** (a) AFM image of an In_2_O_3_ pattern (40 μm × 80 μm). Scale bar: 10 μm. (b) Average thickness of In_2_O_3_ patterns on silicon and UTG substrates versus pulse number.

**Figure S19.** AFM images of In_2_O_3_ films on silicon and UTG substrates after different numbers of pulses.

XPS is employed to investigate the composition and bonding states of In_2_O_3_ films processed with different numbers of pulses on silicon (Figure S20a) and UTG (Figure S20b) substrates. The relative proportions of the oxygen states are summarized in Figure S20c. With increasing pulse number, the fraction of M-O-M bonds rises on both silicon and UTG substrates.

**Figure S20.** (a) XPS O1s spectra of In_2_O_3_ films on silicon substrates after different numbers of pulses. (b) XPS O1s spectra of In_2_O_3_ films on UTG substrates after different numbers of pulses. (c) Relative contributions of M-O-M bonds, oxygen vacancies, and M-OH groups as a function of pulse number.

XRD analysis is employed to examine the crystallinity of the In_2_O_3_ films. XRD patterns of In_2_O_3_ on silicon substrates show distinct diffraction peaks, whose intensity gradually increases with pulse number (Figure S21a). In contrast, no obvious diffraction peaks are observed for In_2_O_3_ films on UTG substrates (Figure S21b).

**Figure S21.** (a) XRD patterns of In_2_O_3_ films on silicon substrates annealed with different numbers of pulses. (b) XRD patterns of In_2_O_3_ films on UTG substrates annealed with different numbers of pulses.

**Note 13.** **Structural layout and fabrication process flow of the MOTFTs.**

The fabricated MOTFT consists of In_2_O_3_ as channel layer, ITO as source/drain and gate electrodes, and Al_2_O_3_ as gate insulator layer. Figure S22 presents schematic diagrams of the step-by-step fabrication process of the TFTs along with corresponding optical micrographs of the patterns. First, a semiconducting In_2_O_3_ channel layer measuring 40 μm × 80 μm is fabricated using the SEDA technique combined with FLA treatment. It should be specifically noted that the source and drain regions are precisely aligned with the first channel layer. ITO source/drain electrodes with a width of 100 μm and a spacing of 20 μm are fabricated employing the SEDA technique combined with FLA treatment. Similarly, the Al_2_O_3_ (100 μm × 160 μm) gate dielectric layer and the ITO gate electrode are sequentially fabricated via the SEDA process and FLA treatment, ultimately achieving the integrated fabrication of the TFTs.

**Figure S22.** The schematic illustrations of the MOTFT fabrication process and optical micrographs of the devices on silicon and UTG substrates. Scale bar: 50 μm.

**Note 14.** **Transfer characteristic of MOTFTs under 20-pulse annealing.**

Figure S23 shows the transfer characteristic of MOTFTs under 20-pulse annealing. The leakage current of the gate dielectric layer reaches the order of tens of microamperes, leading to device failure.

**Figure S23.** Transfer characteristic of MOTFTs under 20-pulse annealing.

**Note 15.** **Comparison of the electrical performance of MOTFTs in this work and in the literature.**

Figure S24 shows the transfer characteristics of MOTFTs processed by FLA (100 pulses) and by CTA (400 ℃, 1 h). The FLA-processed TFT exhibits a field‑effect mobility of 8.04 cm^2^ V^-1^ s^-1^, a threshold voltage of 0.22 V, and an on/off ratio of 10^8^. In contrast, The CTA-processed TFT shows a mobility of 1.86 cm^2^ V^-1^ s^-1^, a threshold voltage of 2.95 V, and an on/off ratio of 10^6^. The FLA-processed TFT thus demonstrates superior electrical performance while reducing the processing time by up to two orders of magnitude.

**Figure S24.** Comparison of transfer characteristics for MOTFTs fabricated by FLA and CTA.

The electrical performance of the TFTs presented in this work is benchmarked against previously reported metal‑oxide TFTs. Table S7 presents a comprehensive compilation of the electrical performance metrics, including mobility (*μ*), threshold voltage (*V*_th_), subthreshold swing (*SS*), and leakage current (*I*_leakage_). These TFTs, each with a defined channel width (*W*) and channel length (*L*), have been fabricated using various techniques such as the SEDA process introduced here, inkjet printing, spin coating, and sputtering. Different materials are used for the gate electrode (G), gate insulator (GI), semiconductor channel, and source/drain electrodes (SD). The TFTs also employ a range of annealing methods to enhance performance, including the FLA process utilized in this study, along with conventional thermal annealing (CTA), rapid thermal annealing (RTA), deep‑ultraviolet (DUV) annealing, far‑ultraviolet (FUV) annealing, laser annealing (LA), and near‑infrared (NIR) annealing.

**Table S7.** Summary of electrical performance parameters for MOTFTs from this work and the literature.

|  | **FLA-processed layers** | **Fabrication techniques** | **Materials**  **(G/GI/Channel/SD)** | | **Annealing parameters** | ***W***  **(μm)** | ***L***  **(μm)** | ***μ***  **(cm^2^ V^-1^ s^-1^)** | | ***V*_th_ (V)** | ***SS* (V/decade)** | | | ***I*_leakage_**  **(A)** | **Ref.** |
| --- | --- | --- | --- | --- | --- | --- | --- | --- | --- | --- | --- | --- | --- | --- | --- |
|  | N/A | Inkjet | ITO/Al_2_O_3_/SnO_2_/ITO | CTA (350 ℃, 1 h) | | 600 | 30 | 13.3 | 0.14 | | | 0.13 | 10^-9^ | | [39] |
|  | N/A | Inkjet | Al/Al_2_O_3_/In_2_O_3_/Al | CTA (300 ℃, 0.5 h) and FUV | | 1000 | 80 | 4.3 | 1.70 | | | - | - | | [40] |
|  | N/A | Inkjet | ITO/ZrO_2_/IGO/ITO | CTA (450 ℃, 1 h) | | 60 | 40 | 7.5 | 0.04 | | | 0.14 | 10^-11^ | | [41] |
|  | N/A | SEDA | Si/Al_2_O_3_/IGZO/Al | CTA (385 ℃, 1 h) | | 500 | 50 | 5.3 | 0.80 | | | 0.10 | 10^-9^ | | [42] |
|  | N/A | SEDA | ITO/Al_2_O_3_/In_2_O_3_/ITO | CTA (400 ℃, 1 h) | | 80 | 20 | 6.3 | 1.76 | | | 0.34 | 10^-11^ | | [5] |
|  | N/A | Spin | Si/SiO_2_/ WZTO/Al | LA | | 700 | 70 | 4.99 | 4.13 | | | 0.54 | - | | [43] |
|  | N/A | Spin | Si/SiO_2_/Si_3_N_4_/ IGZO/Al/Ti | RTA | | 80 | 20 | 0.78 | -2.8 | | | 0.85 | - | | [44] |
|  | Channel | Sputtering | Si/SiO_2_/ZnON/Al | FLA | | 800 | 200 | 48.4 | 0.98 | | | 0.26 | - | | [45] |
|  | Channel | Spin | Tiw/ Al_2_O_3_/HfO_2_/IZO/Al | FLA (10 min) | | 20 | 10 | 12.52 | -5.68 | | | 0.29 | 10^-10^ | | [46] |
|  | Channel | Spin | Mo/HfO_2_/IZO/Al | FLA (50 s) | | 500 | 60 | 21.8 | -7.3 | | | 0.28 | 10^-9^ | | [47] |
|  | Channel | Spin | Al/ Al_2_O_3_/IGZO/Au/ITO | FLA (17 s) | | 500 | 4 | 3.56 | - | | | 0.08 | 10^-8^ | | [48] |
|  | Channel | Spin | Al/ Al_2_O_3_/ ZrO_2_/ In_2_O_3_/Al | FLA (17 s) | | 1000 | 40 | 6 | 0.6 | | | - | - | | [49] |
|  | Channel | Spin | Si/SiO_2_/ IGZO/Al | FLA, DUV and NIR | | 500 | 50 | 7.7 | - | | | 0.44 | - | | [50] |
|  | Channel | Spin | Si/SiO_2_/IGZO/Ag | FLA, DUV and NIR | | 500 | 70 | 7.96 | - | | | - | - | | [51] |
|  | Channel | Spin | Si/SiO_2_/ In_2_O_3_/Al | FLA+DUV | | 100 | 1000 | 5.35 | ~0 | | | - | - | | [52] |
| GI and Channel | | Spin | Si/Al_2_O_3_/IGZO/Al | FLA (390 s) | | 1500 | 200 | 2.99 | 0.73 | | | 0.18 | 10^-10^ | | [53] |
| GI and Channel | | Spin | Al/ Al_2_O_3_/ ZrO_2_/ In_2_O_3_/Al | FLA | | - | - | 5.7 | 0.73 | | | - | 10^-7^ | | [54] |
|  | GI and Channel | Spin | ITO/ZrO_2_/ZnO/Al | FLA | | *W*/*L*=6:1 | | 3.4 | 0.8 | | | 0.3 | - | | [55] |
|  | Channel | SEDA | Si/SiO_2_/ In_2_O_3_/Al | FLA (5 s) | | 80 | 20 | 2.19 | 3.21 | | | 0.47 | 10^-11^ | | This  work |
|  | All layers | SEDA | ITO/Al_2_O_3_/In_2_O_3_/ITO | FLA (100 s) | | 80 | 20 | 7.59 | 0.10 | | | 0.40 | 10^-11^ | | This  work |

**Note 16.** **Stability test of the electrical performance of MOTFTs** **fabricated via FLA.**

The electrical stability of the MOTFTs is investigated using the positive bias stress (PBS) test. During the test, a constant gate bias (*V*_bias_) is applied between the gate and source electrodes. The MOTFT fabricated on the silicon substrate demonstrates high PBS stability, with a minimal threshold voltage shift of 0.08 V and a drain current reduction of 5.7% over a period of 3600 s. In contrast, the MOTFT fabricated on UTG exhibits inferior PBS stability. The threshold voltage shifts by 0.34 V after 3600 s of stress, which is larger than that observed for the MOTFT on silicon. In addition, a higher drain current reduction of 22.3% is observed. The positive threshold voltage shift for both MOTFTs is attributed to electron trapping at the In_2_O_3_/Al_2_O_3_ interface. The reduction in drain current and the enhanced positive shift in threshold voltage for the MOTFT on UTG can be attributed to the higher hydroxyl (-OH) group content in the Al_2_O_3_ gate dielectric on UTG. These hydroxyl groups act as electron traps, capturing electrons within the In_2_O_3_ channel and thereby requiring a larger gate voltage to induce sufficient channel carriers. This results in a suppressed drain current and an enhanced positive shift of the threshold voltage.

**Figure S25.** Electrical stability test of the flash‑lamp‑annealed MOTFTs on both silicon and UTG substrates. (a) Transfer curves of one MOTFT on the silicon substrate during the PBS stability test. (b) Dependence of the threshold voltage shift on the stress time for the MOTFT on the silicon substrate. (c) Transfer curves of one MOTFT on the UTG substrate during the PBS stability test. (d) Dependence of the threshold voltage shift on the stress time for the MOTFT on the UTG substrate.

The environmental stability of the flash-lamp-annealed MOTFTs on both silicon and UTG substrates is evaluated after six months of storage in ambient air. Figure S26 presents the transfer curves of the MOTFTs before and after this storage period. The extracted parameters—including field-effect mobility ($\mu$), threshold voltage (*V*_th_), and subthreshold swing (*SS*)—exhibit only minor variations, thereby confirming the excellent environmental stability of the MOTFTs.

**Figure S26.** Environmental stability test of the flash‑lamp‑annealed MOTFTs on both silicon and UTG substrates. (a) Transfer curves of one MOTFT on the silicon substrate before and after 6 months of storage in ambient air. (b) Field-effect mobility ($\mu$), threshold voltage (*V*_th_), and subthreshold swing (*SS*) of the MOTFT on the silicon substrate before and after 6 months of storage in ambient air. (c) Transfer curves of one MOTFT on the UTG substrate before and after 6 months of storage in ambient air. (d) Field effect mobilities, threshold voltages and subthreshold swings of the MOTFT on the UTG substrate before and after 6 months of storage in ambient air.

Figure S27a schematically illustrates the bending geometry used in the mechanical stability test. The UTG substrate with a thickness of *t*=145 µm is bent to a radius of *R*_1_=45 mm. The surface strain ($\varepsilon$) is determined by the neutral plane of the bent substrate, yielding a value of approximately 0.2% based on the expression:

$\varepsilon=\frac{R_{1}\theta-r\theta}{r\theta}=\frac{R_{1}-r}{r}=\frac{t/2}{R_{1}-t/2}$	(S31)

where *r* is the radius of curvature of the neutral surface, and $\theta$ is the bending angle. It should be noted that this bending radius (*R*_1_=45 mm) is the minimum radius that the 145‑µm‑thick UTG can withstand in practical testing—further reducing the bending radius would cause the UTG to fracture in the test fixture, preventing it from maintaining the intended bending geometry. To further assess the mechanical stability of the MOTFTs fabricated on UTG substrates treated with FLA, one MOTFT is subjected to cyclic bending at a radius of 45 mm for up to 1000 cycles, with transfer characteristics recorded every 200 cycles, as shown in Figure S27b. A comparison of the transfer curves across different cycle numbers reveals that they are virtually superimposable. Furthermore, the field-effect mobility ($\mu$), threshold voltage (*V*_th_), and subthreshold swing (*SS*) exhibit no apparent degradation with increasing cycle number, confirming the excellent mechanical stability of the MOTFT.

**Figure S27.** Bending stability test for MOTFT. (a) Schematic illustration of the setup to generate tensile strain at the outer surface of the UTG substrate. (b) Transfer characteristics of one MOTFT fabricated on the UTG substrate under cyclic bending at a radius of 45 mm for up to 1000 cycles.

**Note 17.** **Comprehensive characterization of key electronic and optical properties: work function (*Φ*), energy difference between the valence band maximum and the Fermi level (*E*_VBM-F_), and optical bandgap (*E*_g_) for both In_2_O_3_ and ITO.**

The optical bandgap (*E*_g_) is quantified by analyzing the absorption coefficient obtained with a UV-vis spectrometer (Figure S28). *E*_g_ is extracted from the absorption spectra using the Tauc relation:

(𝛼h𝜈)^2^ = A(h𝜈 − *E*_g_)	(S32)

where h is the Planck constant, and *v* is the photon frequency, 𝛼 is the absorption coefficient, and A is a constant.

**Figure S28.** Tauc plots for determining the bandgap of ITO (a) and In_2_O_3_ (b) films processed with varying pulse numbers on UTG substrates.

To obtain the band diagrams of ITO and In_2_O_3_, UPS analysis is performed on both materials processed using FLA with 100 pulses. Figure S29a presents the UPS spectra of the secondary electron edge for ITO and In_2_O_3_ films. The secondary electron cutoff energy (*E*_cutoff_) is estimated by finding the intersection point of the linear extrapolation of the spectral slope with the baseline. The work function (*Φ*) is then calculated by subtracting *E*_cutoff_ from the UV photon energy (*hv* = 21.22 eV):

$\Phi=hv-E_{cutoff}$	(S33)

Figure S29b displays the UPS spectra in the valence band region for the ITO and In_2_O_3_ films. The energy difference between the valence band maximum and Fermi energy level (*E*_VBM-F_) is determined by the intersection of the linear extrapolation of the spectral slope with the baseline.

**Figure S29.** (a) UPS spectra of the secondary electron edge for In_2_O_3_ and ITO films. (b) UPS spectra at the valence band region for In_2_O_3_ and ITO films.

**Table S8.** Work function (*Φ*), energy difference between the valence band maximum and the Fermi level (*E*_VBM-F_), and optical bandgap (*E*_g_) for In_2_O_3_ and ITO.

| Metal oxides | *Φ* (eV) | *E*_VBM-F_ (eV) | *E*_g_ (eV) |
| --- | --- | --- | --- |
| In_2_O_3_ | 4.41 | 3.14 | 3.75 |
| ITO | 4.38 | 3.28 | 3.77 |

**Note 18. Long-term stability of all‑solution‑processed logic circuits fabricated via FLA.**

Logic circuits composed of all‑solution‑processed MOTFTs treated with FLA exhibit exceptional long‑term stability, as presented in Figure S30. After six months of ambient storage, the input-output responses of all logic gates are re‑evaluated. Every gate (NOT, NOR, NAND, and AND) retains its correct logic functionality, with negligible degradation in output signal amplitude or waveform, underscoring the outstanding resilience of the FLA‑processed all‑solution devices against environmental aging under prolonged atmospheric exposure.

**Figure S30.** Post‑storage logic operation of the circuits after six months in air. (a) Input signal 1 (*V*_IN1_). (b) Input signal 2(*V*_IN2_). (c) NOT‑gate operation under *V*_IN1_. (d-f) Logic responses of the NOR, AND, and NAND gates, respectively, under *V*_IN1_ and *V*_IN2_.

**Note 19.** **Dynamic response of logic gate circuits to square-wave input signals.**

The inverter fabricated with all-solution-processed TFTs treated with FLA demonstrates excellent dynamic response characteristics, as shown in Figure S31. For dynamic testing, all electrical connections are made via probes. A signal generator supplies rectangular input waves with an amplitude of ± 2 V at various frequencies, while a semiconductor parameter analyzer provided a supply voltage (*V*_DD_) of 1 V. The output signals are recorded using an oscilloscope. The inverter achieves nearly delay‑free inversion at 1 kHz and retains clear signal inversion functionality up to 4 kHz.

**Figure S31.** Dynamic response of the inverter to rectangular input waves (±2 V amplitude) at different frequencies, with *V*_DD_ = 1 V.

**Note 20.** **Schematic illustration of the fabrication of functionalized substrates.**

An oxygen plasma treatment is employed to clean substrates and enhance their surface reactivity. A trimethoxy(octyl)silane SAM film is deposited on the substrate using a chemical vapor deposition process to render the entire surface hydrophobic. Subsequently, the substrate is selectively irradiated through a photomask with vacuum ultraviolet (VUV) light, rendering the exposed regions hydrophilic while the unexposed areas remain hydrophobic. The patterned hydrophilic regions possess high surface energy, whereas the hydrophobic non-pattern regions maintain low surface energy, thereby forming a functionalized substrate with surface energy contrast patterning.

**Figure S32.** Schematic illustration of the fabrication of functionalized substrates.

**References**

[1]	Y. Kim, S. Park, S. Kim, B.-K. Kim, Y. Choi, J.-H. Hwang, H. J. Kim, *Thin Solid Films* **2017**, 628, 88.

[2]	J. B. Plumley, A. W. Cook, C. A. Larsen, K. Artyushkova, S. M. Han, T. L. Peng, R. A. Kemp, *Journal of Materials Science* **2018**, 53, 12949.

[3]	E. Gilshtein, S. Bolat, G. T. Sevilla, A. Cabas-Vidani, F. Clemens, T. Graule, A. N. Tiwari, Y. E. Romanyuk, *Advanced Materials Technologies* **2020**, 5, 2000369.

[4]	K. Daoudi, B. Canut, M. G. Blanchin, C. S. Sandu, V. S. Teodorescu, J. A. Roger, *Materials Science and Engineering: C* **2002**, 21, 313.

[5]	J. Zhang, G. Wang, Z. Chai, Z. Li, S. Yuan, Y. Wang, Y. Ding, T. Sun, T. Wang, D. Zhao, A. A. Busnaina, T.-L. Ren, X. Lu, *ACS Nano* **2024**, 18, 20493.

[6]	A. Zavaliangos, J. Zhang, M. Krammer, J. R. Groza, *Materials Science and Engineering: A* **2004**, 379, 218.

[7]	R. E. Sanders Jr, S. Updated by, in *Kirk‐Othmer Encyclopedia of Chemical Technology* **2012**, p. 1.

[8]	E. Bartonickova, J. Svec, L. Dlabajova, P. Hruby, V. Caba, M. Sedlacik, L. Kalina, J. Koplik, F. Soukal, L. Kersnerova, J. Zach, V. Novak, K. Dvorak, *Ceramics International* **2025**, 51, 5505.

[9]	G. C. Righini, M. Ferrari, A. Lukowiak, G. Macrelli, *Materials* **2025**, 18.

[10]	A. Masolin, P.-O. Bouchard, R. Martini, M. Bernacki, *Journal of Materials Science* **2012**, 48, 979.

[11]	T. Ashida, A. Miyamura, N. Oka, Y. Sato, T. Yagi, N. Taketoshi, T. Baba, Y. Shigesato, *Journal of Applied Physics* **2009**, 105, 073709.

[12]	A. Minenkov, S. Hollweger, J. Duchoslav, O. Erdene-Ochir, M. Weise, E. Ermilova, A. Hertwig, M. Schiek, *ACS Applied Materials & Interfaces* **2024**, 16, 9517.

[13]	J. M. Davies, W. Zagieboylo, *Appl. Opt.* **1965**, 4, 167.

[14]	M. R. Querry, 1985.

[15]	K. M. McPeak, S. V. Jayanti, S. J. P. Kress, S. Meyer, S. Iotti, A. Rossinelli, D. J. Norris, *ACS Photonics* **2015**, 2, 326.

[16]	J. Chen, A. Riaz, M. Taheri, A. Kumar, J. Coventry, W. Lipiński, *Journal of Quantitative Spectroscopy and Radiative Transfer* **2021**, 272.

[17]	D. Franta, A. Dubroka, C. Wang, A. Giglia, J. Vohánka, P. Franta, I. Ohlídal, *Applied Surface Science* **2017**, 421, 405.

[18]	S. Y. El-Zaiat, *Optik* **2013**, 124, 157.

[19]	B. Harbecke, *Applied Physics B* **1986**, 39, 165.

[20]	S. Zhao, X. Sun, Z. Li, W. Xie, S. Meng, C. Wang, W. Zhang, *Applied Thermal Engineering* **2019**, 146, 569.

[21]	N. Daouas, *Numerical Heat Transfer, Part B: Fundamentals* **2018**, 73, 106.

[22]	B. Mondal, S. C. Mishra, *Numerical Heat Transfer, Part A: Applications* **2007**, 52, 757.

[23]	B. Majaron, M. Milanič, *Physics in Medicine and Biology* **2008**, 53, 255.

[24]	A. Heimsath, P. Nitz, *Solar Energy Materials and Solar Cells* **2019**, 203, 110191.

[25]	F. Cadiz, A. Couairon, in *Classical Electrodynamics: Fundamentals and Applications*, Springer **2025**, p. 549.

[26]	N. Khatoon, B. Subedi, D. B. Chrisey, *ChemistryOpen* **2024**, 13, e202300260.

[27]	H. R. Shanks, P. D. Maycock, P. H. Sidles, G. C. Danielson, *Physical Review* **1963**, 130, 1743.

[28]	C. J. Glassbrenner, G. A. Slack, *Physical Review* **1964**, 134, A1058.

[29]	V. I. Brinzari, A. I. Cocemasov, D. L. Nika, G. S. Korotcenkov, *Applied Physics Letters* **2017**, 110, 071904.

[30]	Y. Hirata, Y. Kinoshita, T. Shimonosono, T. Chaen, *Ceramics International* **2017**, 43, 9973.

[31]	R. Barea, M. I. Osendi, J. M. F. Ferreira, P. Miranzo, *Acta Materialia* **2005**, 53, 3313.

[32]	M. W. Chase, *Journal of physical and chemical reference data* **1998**, 28, 1951.

[33]	C.-L. Tien, T.-W. Lin, *Coatings* **2021**, 11, 153.

[34]	J. Kim, S. Shrestha, M. Souri, J. G. Connell, S. Park, A. Seo, *Scientific Reports* **2020**, 10, 12486.

[35]	Y. Okada, Y. Tokumaru, *Journal of Applied Physics* **1984**, 56, 314.

[36]	G. E. Jellison, Jr., F. A. Modine, *Journal of Applied Physics* **1994**, 76, 3758.

[37]	M. A. Green, *Solar Energy Materials and Solar Cells* **2008**, 92, 1305.

[38]	H. Schneider, J. Schreuer, B. Hildmann, *Journal of the European Ceramic Society* **2008**, 28, 329.

[39]	K. Liang, H. Ren, D. Li, Y. Wang, Y. Tang, M. Zhao, H. Wang, W. Li, B. Zhu, *Journal of Materials Chemistry C* **2021**, 9, 11662.

[40]	J. Leppäniemi, K. Eiroma, H. Majumdar, A. Alastalo, *ACS Applied Materials & Interfaces* **2017**, 9, 8774.

[41]	Y. Li, L. Lan, S. Sun, Z. Lin, P. Gao, W. Song, E. Song, P. Zhang, J. Peng, *ACS Applied Materials & Interfaces* **2017**, 9, 8194.

[42]	W.-J. Lee, W.-T. Park, S. Park, S. Sung, Y.-Y. Noh, M.-H. Yoon, *Advanced Materials* **2015**, 27, 5043.

[43]	M. Xu, C. Peng, Y. Yuan, X. Li, J. Zhang, *ACS Applied Electronic Materials* **2020**, 2, 2970.

[44]	K.-S. Kim, S.-W. Lee, S.-M. Oh, W.-J. Cho, *Materials Science and Engineering: B* **2013**, 178, 811.

[45]	H.-J. Jeong, H.-M. Lee, C.-H. Ryu, E.-J. Park, K.-L. Han, H.-J. Hwang, K.-C. Ok, H.-S. Kim, J.-S. Park, *ACS Applied Materials & Interfaces* **2019**, 11, 4152.

[46]	Y. F. Abed, N. Chatterjee, B. Luo, S. L. Swisher, *Advanced Engineering Materials* **2025**, 27, 2403042.

[47]	A. M. Weidling, V. S. Turkani, B. Luo, K. A. Schroder, S. L. Swisher, *ACS Omega* **2021**, 6, 17323.

[48]	L. Luo, H. Faber, C. Liu, S. Doukas, E. Yarali, B. Adilbekova, D. R. Naphade, N. Xiao, Y. Ma, H. F. Mazo‐Mantilla, L. Panagiotidis, W. S. Alghamdi, C. F. Florica, M. I. Nugraha, X. Li, X. Zhang, M. Heeney, E. Lidorikis, T. D. Anthopoulos, *Advanced Functional Materials* **2024**, 34, 2406044.

[49]	K. Tetzner, Y.-H. Lin, A. Regoutz, A. Seitkhan, D. J. Payne, T. D. Anthopoulos, *Journal of Materials Chemistry C* **2017**, 5, 11724.

[50]	C.-J. Moon, H.-S. Kim, *ACS Applied Materials & Interfaces* **2019**, 11, 13380.

[51]	C.-J. Moon, J.-W. Park, Y.-R. Jang, H.-S. Kim, *Scientific Reports* **2024**, 14, 1566.

[52]	J. Shin, J. Kim, S. Park, S. J. Kim, J. A. Lim, S. Ko, *Organic Electronics* **2022**, 104, 106476.

[53]	Y.-W. Oh, H. Kim, L.-M. Do, K.-H. Baek, I.-S. Kang, G.-W. Lee, C.-m. Kang, *RSC Advances* **2024**, 14, 37438.

[54]	E. Yarali, H. Faber, E. Yengel, A. Seitkhan, K. Loganathan, G. T. Harrison, B. Adilbekova, Y. Lin, C. Ma, Y. Firdaus, T. D. Anthopoulos, *Advanced Electronic Materials* **2020**, 6, 2000028.

[55]	S. K. Sarkar, D. Maji, J. A. Khan, S. Kurup, D. Gupta, *ACS Applied Electronic Materials* **2022**, 4, 2442.
